# Discrimination of *Dendropanax morbifera* via HPLC fingerprinting and SNP analysis and its impact on obesity by modulating adipogenesis- and thermogenesis-related genes

**DOI:** 10.3389/fnut.2023.1168095

**Published:** 2023-08-02

**Authors:** Muhammad Awais, Reshmi Akter, Vinothini Boopathi, Jong Chan Ahn, Jung Hyeok Lee, Ramya Mathiyalagan, Gi-Young Kwak, Mamoona Rauf, Deok Chun Yang, Geun Sik Lee, Yeon-Ju Kim, Seok-Kyu Jung

**Affiliations:** ^1^Graduate School of Biotechnology, College of Life Sciences, Kyung Hee University, Yongin si, Republic of Korea; ^2^Department of Botany, Abdul Wali Khan University Mardan, Mardan, Pakistan; ^3^Department of Oriental Medicinal Biotechnology, College of Life Sciences, Kyung Hee University, Yongin-si, Republic of Korea; ^4^Southwest Coast Hwangchil Cooperative, Chonnam National University, Gwangju si, Republic of Korea; ^5^Jungwon University Industry Academic Cooperation Building, Goesan-gun, Republic of Korea; ^6^Department of Horticulture, Kongju National University, Yesan, Republic of Korea

**Keywords:** plant secondary metabolites, chlorogenic acid, rutin, multiplex PCR, *in silico*, *in vitro*

## Abstract

*Dendropanax morbifera* (DM), a medicinal plant, is rich in polyphenols and commonly used to treat cancer, inflammation, and thrombosis. However, to date, no study has been conducted on DM regarding the enormous drift of secondary metabolites of plants in different regions of the Republic of Korea and their effects on antiobesity, to explore compounds that play an important role in two major obesity-related pathways. Here, we present an in-depth study on DM samples collected from three regions of the Republic of Korea [Jeju Island (DMJ), Bogildo (DMB), and Jangheung (DMJG)]. We used high-performance liquid chromatography (HPLC) and multivariate component analyses to analyze polyphenol contents (neochlorogenic acid, chlorogenic acid, cryptochlorogenic acid, and rutin), followed by discrimination of the samples in DMJG using single nucleotide polymorphism and chemometric analysis. *In silico* and *in vitro* evaluation of major compounds found in the plant extract on two major anti-obesity pathways (adipogenesis and thermogenesis) was carried out. Furthermore, two extraction methods (Soxhlet and ultrasound-assisted extraction) were used to understand which method is better and why. Upon quantifying plant samples in three regions with the polyphenols, DMJG had the highest content of polyphenols. The internal transcribed region (ITS) revealed a specific gel-based band for the authentication of DMJG. PCA and PLS-DA revealed the polyphenol’s discriminative power of the region DMJG. The anti-obesity effects of plant extracts from the three regions were related to their polyphenol contents, with DMJG showing the highest effect followed by DMJ and DMB. Ultrasound-assisted extraction yielded a high number of polyphenols compared to that of the Soxhlet method, which was supported by scanning electron microscopy. The present work encourages studies on plants rich in secondary metabolites to efficiently use them for dietary and therapeutic purposes.

## 1. Introduction

*Dendropanax morbifera* (DM), an evergreen tree commonly known as Hwangchil, belongs to the family Araliaceae. DM is golden in color and medium-sized, approximately 15 meters in height, with leaves with a duck-feet composition. DM produces black fruits between September and November and grows in the Republic of Korea. The genus *Dendropanax* has the maximum species diversity, consisting of 91–95 species distributed across China, the Republic of Korea, Malaysia, Vietnam, Japan, Thailand, Taiwan, Laos, Mexico, Central America, Colombia, Peru, Bolivia, Venezuela, and Brazil (1). Several parts of DM, including edible leaves, seeds, bark, and roots, are used as alternative traditional medicines and food additives and are registered with the Ministry of Food and Drug Safety, Korea Food and Drug Administration^1^ (1).

DM grows in warm and wet tropical regions of the Republic of Korea, such as Jeju Island, Bogildo, Jangheung, Wando Island, Kangjin, Haenam, and Yeosu. In recent years, studies on DM to analyze its efficacy are being conducted *in vitro* and *in vivo* as it is rich in plant secondary metabolites (PSMs). However, little attention has been given to PSMs found in DM and their variability across different regions in the Republic of Korea. PSMs are chemical compounds that are abundantly produced by plant cells through metabolic pathways and do not directly influence the growth and normal function of plants (2); however, PSMs show various biological effects in response to microbes and play a role in the environmental adaptation of plants (3). DM contains polyphenols, flavonoids, essential oils, tannins, and alkaloids. Some of the primary polyphenols include chlorogenic acid (CGA), neochlorogenic acid (NCGA), cryptochlorogenic acid (CCGA), and rutin (4). The variability of PSMs in DM on a seasonal basis or with different parts in DM has been reported in a few studies. Youn et al. (5) collected samples in May, August, and November, and applying water with 30% and 60% ethanol revealed that the samples in May provided CGA and rutin of high quantity with 60% ethanol. In contrast, irregularity of CGA and rutin in different regions of the Republic of Korea (Wando, Kangjin, DMJ, and DMJG) in DM has been reported by Choi et al. (6). However, chemical and genetical discrimination of plants with high amounts of PSMs in the Republic of Korea has been unsuccessful.

Plants exhibit varying levels of PSMs in different geographical regions; they serve as bioactive components aiding plant adaptation to specific environmental conditions, and their distribution among plant organs is influenced by biotic and abiotic factors (7). The presence of varied amounts of PSMs affects the efficacy of plant extracts on animal models. Obesity, a common metabolic disorder, is characterized by adipocyte hypertrophy owing to an imbalance between food intake and energy expenditure (8). It is associated with other metabolic dysfunctions such as insulin resistance, non-alcoholic fatty liver disease, type-2 diabetes, dyslipidemia, coronary disorder, hypertension, and cancer. The enlargement of adipose tissue causes fat storage in existing adipocytes and converts preadipocytes into mature adipocytes in a process known as adipogenesis (9). Moreover, brown adipose tissue generates heat through a process called thermogenesis to keep the body warm in low-temperature conditions or to waste food energy. It contributes to energy and temperature balance, and obesity results when this does not function properly. Therefore, controlling adipogenesis and inducing thermogenesis are indispensable to prevent obesity. Plants and their metabolites prevent obesity by inhibiting adipogenesis and stimulating lipolysis (10). Song et al. (11) reported that water extract from DM leaves shows anti-obesity potential by inhibiting adipogenesis in 3T3-L1 cells; however, they could not identify particular compounds responsible for such effects. Moreover, thermogenesis-related genes were not considered, which play a vital role in anti-obesity.

Studies on medicinal plants primarily focus on PSMs, particularly when plant extracts are used on animal cell lines or *in vivo*. Several methods have been designed for isolating the secondary compounds in high contents from plants including conventional (Soxhlet) and modern [ultrasound-assisted extraction (UAE)] methods. Furthermore, solvent ratio, quantity of plant material, time, extraction temperature, and other factors are considered. A prominent method for this purpose is response surface methodology (RSM). RSM maximizes extraction yield in a low number of trials by determining the quadruple effect of a factor or interaction between several factors to acquire a high-precision prediction of an optimum value (12). The techniques used and factors involved in the isolation of plant compounds are still debatable. Zhang et al. (13) optimized conditions for isolating caffeic acid from DM using RSM. Eom et al. (14), using RSM, analyzed the optimal effects of total flavonoids, ferric reducing antioxidant power, and Trolox equivalent antioxidant capacity on alcohol-induced liver injury. However, existing reports on DM provide no clue or use one of the two methods for isolating PSMs.

Here we provide detailed information on the distribution of PSMs (CGA, NCGA, CCGA, and rutin) in three regions of the Republic of Korea. Firstly, we do this by comparing Soxhlet and UAE extraction methods, which process yields high bioactive components, before debating and discussing the possible mechanisms. Furthermore, we used single nucleotide polymorphism (SNP) and chemotactic analysis for the discrimination of plants and simultaneous identification of CGA, NCGA, CCGA, and rutin. We performed *in silico* analysis underlining the major compounds in DM, which are responsive to the two major anti-obesity pathways (adipogenesis and thermogenesis), and compared the anti-obesity efficacy of DM from three regions using the 3T3-L1 cell lines for the first time.

## 2. Materials and methods

### 2.1. Plant experiment

#### 2.1.1. Sample collection and processing

A total of 10 batches of 12-years-old DM from DMJG (34°45′36.6”N 126°53′55.5″E), DMJ (33°22′34.3”N 126°49′51.3″E), and DMB (34°08′58.9”N 126°32′35.7″E) in the Republic of Korea were obtained in July 2021. The samples were washed with tap water to remove any residue and then dried at 35°C for 20 h in a L’EQUIP dehydrator.

#### 2.1.2. Chemicals and materials

Four reference compounds, NCGA, CGA, CCGA, and rutin, were purchased from ChemFaces (Wuhan, Hubei, China). HPLC-grade water, acetonitrile, and analytical-grade phosphoric acid were acquired from Honeywell (Republic of Korea).

#### 2.1.3. Sample extraction for HPLC analysis and RSM

Dried samples were ground for approximately 5 min using a Chuhen grinder (1500 W, model number CM-PC100DS) to obtain fine powder. For extraction of PSMs, percentage of ethanol, solvent-to-sample ratio, and time were applied according to RSM software (Design Expert 12). For both Soxhlet and UAE methods, we applied the same conditions that were generated using RSM. We underlined the lower, middle, and upper levels of the three factors. The solvent/plant ratio, time, and extraction temperature were 20, 30, and 40 mL/g; 100, 140, and 180 min; and 40, 60, and 80°C, respectively, which were considered based on the values obtained in preliminary experiments. The total number of experimental runs was 17. Out of the 17 tests, we selected only one condition that resulted in the highest amounts of extracted polyphenols. Detailed information on RSM conditions is provided in Supplementary Table S1.

#### 2.1.4. Morphology of plant extract

After extraction, the samples were dried and analyzed using scanning electron microscopy (SEM, Hitachi Tabletop Microscope TM-1000, Japan). For good conduction, the samples were coated with platinum using Quorum (Q150R S).

#### 2.1.5. HPLC conditions

The HPLC system consisted of the Agilent 1260 infinity Quaternary Pump (G1311B), Agilent 1260 infinity Standard Auto Sampler (G1329B), Agilent 1260 Infinity Column Thermostat Compartment (G1316A), and Agilent 1260 Infinity Variable Wavelength Detector (G1314F) as instrumental system, and ZORBAX Eclipse Plus C18 column (250 × 4.6 mm i.d., 5 μm particle size) (Milford, MA, United States) was used as the stationary phase. For simultaneous detection of the four compounds, gradient elution composition was as follows: (0–10 min, 5%–9% channel B; 10–30 min, 9%–9% channel B; 30–60 min, 9%–30% channel B; 60–62 min, 30%–50% channel B; 62–65 min, 50%–5% channel B; and 65–70 min, 5%–5% channel B). Channel A contained 0.4% phosphoric acid in HPLC-grade water, and channel B contained acetonitrile. The wavelength was 327 nm, with a column temperature of 35°C and an injection volume of 5 μL. The four analytical standards and DM plant extract under observation HPLC generated peaks are merged and provided in Supplementary Figure S1.

#### 2.1.6. Method validation of HPLC analysis

For calibration curves, working standard solutions of different concentrations were analyzed by plotting the peak areas of each analyte with respect to its concentration. The limit of detection (LOD) and limit of quantification (LOQ) for each compound were defined as the concentration that produced peaks with signal-to-noise values of 3 and 10, respectively.

We developed an intra-day precision method by repetitively analyzing the sample six times within the same day with a gap of 2 h. For inter-day precision, the sample was examined in triplicates for the following 3 days. In the stability test, the sample was stored at −80°C for a day, brought to room temperature, and analyzed at 0, 2, 4, 6, 8, 10, 12, 24, and 48 h. For confirmation of repeatability, the same sample was extracted in sextuplicate and analyzed independently. Precision, stability, and repeatability were evaluated by relative standard deviations (RSD) of the established method.

### 2.2. Discrimination of plant samples

#### 2.2.1. DNA isolation, and sequence analysis

Genomic DNA was isolated from freshly collected samples using a Plant DNA Extraction kit (Exgene Plant SV mini, GeneAll, Seoul, Republic of Korea) according to the manufacturer’s instructions. The quality and quantity of DNA were determined by measuring the absorbances at 260 and 280 nm using BioTek Synergy HT (VT, United States). For barcoding, three genes, two from chloroplast (*PetD* and *trnL-trnF*) and one from rDNA (ITS), were analyzed to find SNP or insertion and deletion (INDEL) regions. Upon analyzing the sequences using the web tool^2^, *trnL-trnF* was considered unsuitable for designing a specific primer, and *PetD* had no SNPs or INDEL regions. Therefore, ITS was chosen to discriminate the plant samples.

#### 2.2.2. Primer designing

The specific primer designed for the DMJG region was based on an SNP variation in the ITS region. A single nucleotide mismatch was introduced to enhance the specificity of primers. The parameters of the primers were analyzed using a web-based search tool.^3^

#### 2.2.3. Optimized amplification using polymerase chain reaction (PCR) and purification of PCR products

A standard polymerase chain reaction (PCR) with a pair of primers was performed to analyze primer efficiency on gel-based results. PCR was carried out in a 20 μL mixture, which consisted of 10 μL 2× Taq PCR premix (Solgent), 10 ng template DNA, 10 μM each of forward and reverse primers, and 10 μM and 0.6 μM specific primer. The conditions for optimized multiplex PCR involve pre-denaturation for 2 min at 95°C, followed by 36 cycles of 30 s at 95°C for denaturation, and 2 min at 72°C for extension, followed by 6 min at 72°C for final extension. PCR products were analyzed on 1% agarose gel stained with Biofact dye and visualized using ultraviolet light (DNR Bio-Imaging System Mini BIS Pro). For sequencing, the PCR products were purified using a PCR extraction kit (GeneAll, Seoul, Republic of Korea) and sequenced at Genotech Corp. (Daejeon, Republic of Korea).

### 2.3. *In silico* experiment

#### 2.3.1. Molecular docking and ADMET analysis

This study aimed to identify molecular interactions of Peroxisome Proliferator- Activated Receptor-γ (PPARγ) and uncoupling protein 1 (UCP1) with the phytochemicals present in DM. The chemical structures of the phytochemicals and control drugs were obtained from the PubChem (15) database in a Spatial Data File (SDF) format. All molecules were optimized using the Auto Dock tool (16). The crystal structure of the first target PPAR*γ* in a complex with rosiglitazone (PDB ID: 7AWC) (17) was attained from the RCSB-Protein Data Bank. Unfortunately, the three-dimensional (3D) structure of the second target UCP1 was unavailable. In this case, the only possible way was to generate a 3D coordinate of UCP1 by comparative prediction. We built the 3D structure of UCP1 using homology modeling. The amino acid sequence corresponding to UCP1 was obtained from the Universal Protein Resource “UniProt” (accession no: P25874) (18). The obtained PBD structures were preprocessed by removing water molecules and heteroatoms and adding polar hydrogen atoms and required charges. As PPAR*γ* is a widely studied target for obesity, the active site of this target has already been depicted in literature. Therefore, the amino acid residues TYR473, HIS449, SER289, and HIS323, along with some other reported confirmations (19), were considered essential for inhibiting PPARγ. Still, we employed the active site prediction tool, DoGSiteScorer, from the protein plus server (20) to predict the functional binding pocket for both the selected targets. Finally, we used AutoDock Vina (21) to dock ligands and control drugs against the two selected protein targets. Supplementary Table S2 shows the gird sizes for docking. In addition, we employed ADMETlab v.2.0 (22) webserver to predict ADME, physicochemical properties, and toxicity of the compounds used in this study.

### 2.4. *In vitro* experiment

#### 2.4.1. Chemicals

3T3-L1, a preadipocyte cell line obtained from mouse embryo, was purchased from the American Type Culture Collection. High glucose Dulbecco’s Modified Eagle Medium (DMEM) and bovine calf serum (BCS) were obtained from Welgene (Daegu, Republic of Korea). Penicillin–streptomycin was purchased from GeneDEPOT. Cocktails for cell differentiation, human recombinant insulin, 3-isobutyl-1-methylxanthine, and dexamethasone were purchased from Wako (Tokyo, Japan).

#### 2.4.2. Cell culture and differentiation

3T3-L1 cells were cultured in DMEM supplemented with 10% BCS and 1% penicillin–streptomycin and maintained in a humidified incubator at 37°C under 5% CO_2_. For differentiation of preadipocytes, cells were treated with an adipogenesis-inducing medium (MDI) 2 days after post-confluence (day 0). MDI consists of 3-isobutyl-1-methylxanthine, dexamethasone, and insulin. After 72 h (on day 2), cells were stimulated with a maturation medium, which consists of 10 μg/mL insulin with or without the addition of DM extracts. Media was changed every 2 days, followed by incubation for 5–8 additional days. On day 8, fully differentiated adipocytes were viewed using a microscope to observe lipid droplets. Cells cultured in a complete medium only were considered the control.

#### 2.4.3. Cytotoxicity assay

Preadipocytes were seeded in a 96-well plate at a density of 1 × 10^4^ cells/well and kept to attach overnight at 37°C under 5% CO_2_ within an incubator. Cells were treated with DM extracts of different concentrations after discarding old medium, and cells without any treatment were used as the control. After incubation for 24 h, 20 μL 3-[4,5-dimethylthiazol-2-yl]-2,5 diphenyl tetrazolium bromide was added to each well and incubated for 3 h. Finally, 100 μL dimethyl sulfoxide was added to each well. Absorbance was measured at 570 nm using a microplate reader (Bio-Tek Instruments, Inc., Winooski, VT, United States).

#### 2.4.4. Triglyceride measurement

In matured cells lipid droplets were visualized using oil red O to confirm adipogenesis in 3T3-L1 cells following a previously described method (23). Briefly, differentiated cells were washed with 1× phosphate-buffered saline (PBS) and fixed using 4% formalin for 2 h. Cells were then dried and soaked in 60% isopropanol. Cells were stained with oil red O solution for 45 min and washed twice or thrice with distilled water to remove excess dye. Phenotypic changes in fully differentiated cells were captured using an inverted light microscope (Nikon Instruments, Melville, NJ, United States). To quantify triglyceride content (lipid accumulation), 100% isopropanol was added to the mature adipocytes, and absorbance was measured at 520 nm after incubation for 10 min at room temperature (25°C).

#### 2.4.5. RNA isolation and reverse-transcription PCR (RT-PCR)

Total RNA was isolated from mature adipocytes treated with DM extracts using TriZol LS reagent following manufacturer’s instructions (Invitrogen, Carlsbad, CA, United States). Total RNA (1 μg) was used to synthesize cDNA using a cDNA synthesis kit (Onebio, Lithuania, EU) following manufacturer’s instructions. The conditions for cDNA synthesis were 42°C for 1 h followed by 72°C for 5 min. cDNA was used to amplify the targeted genes.

RT-PCR was performed using gene-specific primers listed in Supplementary Table S3. *PPARγ*, *C/EBPα*, and *perilipin* were amplified using the following conditions: 94°C for 30 s for denaturation; annealing at 58°C; and extension at 72°C for 5 min. PCR amplicons were visualized with 1% agarose gel.

### 2.5. Antioxidant assay

#### 2.5.1. Measurement of intracellular reactive oxygen species

To measure reactive oxygen species (ROS) levels, cells were washed with PBS at room temperature and exposed to 10 μM 2′,7′-dichlorofluorescein diacetate (Sigma-Aldrich, St Louis, MO, United States), a common cell-permeable fluorogenic substance. Cells were then incubated for 30 min in the dark at 37°C. Finally, fluorescence was measured between 485 and 495 nm using a Spectra Fluor multi-well fluorescence reader (Tecan, Maninder, Austria).

#### 2.5.2. DPPH scavenging activity assay

Free radical scavenging activity of the plant extracts was assessed using DPPH as previously described by Akter et al. (24). The reaction mixture consisted of 20 μL sample and 180 μL of 0.2 mM DPPH in a 96-well plate in three replicates. The plate was kept in a shaker for 30 min at room temperature in dark. Absorbance was measured at 517 nm using BioTek Synergy HT.

#### 2.5.3. Assay of reducing potential of the plant extracts

Reducing potential was evaluated according to Akter et al. (23). The reaction mixture containing 100 μL samples, 250 μL phosphate buffer, and 250 μL of 1% potassium ferricyanide was incubated at 50°C on a heat block for 20 min. The mixture was cooled, and 250 μL of 10% trichloroacetic acid was added. The mixture was centrifuged at 3,000 rpm for 10 min, and the supernatant was added to 100 μL distilled water and 20 μL of freshly prepared 0.1% ferric acid solution. With three replicates in a 96-well plates, the absorbance was measured at 700 nm using BioTek Synergy HT.

#### 2.5.4. Assay of ABTS radical scavenging activity of the plant extracts

The assay was performed using a commercial kit following manufacturer’s instructions. The sample (10 μL) was mixed with 190 μL ABTS reagent and incubated at room temperature for 6 min. Absorbance was measured at 414 nm using BioTek Synergy HT.

### 2.6. Determination of total phenolic contents

Total phenolic contents of the samples were determined using the Folin–Ciocalteu reagent (23). The plant extract (30 μL) was dissolved in 150 μL of 10% 2 N Folin–Ciocalteu reagent. After 5 min, 160 μL of 7.5% Na_2_CO_3_ was added and incubated in the dark for 1 h. The mixture was poured in a 96-well plate, and absorbance was measured at 715 nm using BioTek Synergy HT. Total phenolic content was assessed from the standard curve using gallic acid as the standard. Results were expressed as μmol gallic acid equivalent per gram dry weight (μmol GAE/g DW).

### 2.7. Data analysis

PCA, PLS-DA, and hierarchical clustering analysis (HCA) were carried out using SIMCA v.14.1 software (Umetric, Umea, Sweden). The extraction conditions used for comparison between UAE and Soxhlet were based on Design Expert 12. All data are expressed as mean ± standard error (SE) of at least three independent experiments. GraphPad Prism (GraphPad Software, La Jolla, CA, United States) was used for statistical analysis. Student’s *t*-test and two-way analysis of variance were used to determine total variations between treated and untreated (control) groups. The difference was considered significant at ^*^p < 0.05, ^**^p < 0.01, and ^***^p < 0.001.

## 3. Result and discussion

### 3.1. Soxhlet and UAE

#### 3.1.1. Extraction of DM using Soxhlet and UAE

Extraction of PSMs from plants is the first crucial step for obtaining the desired secondary metabolites. For the extraction of polyphenols, two of the most frequent methods used are Soxhlet (conventional) and UAE (nonconventional). To maximize the efficiency of extraction, techniques are often compared to search for the best method to achieve high yield from extraction. For instance, Palmieri et al. (25) compared more than two techniques to recover bioactive compounds and compared. In addition, the extraction efficiency of a compound is influenced by multiple parameters, such as temperature and time, and their effects may be independent or interactive (26). All factors can be implemented in a conventional single-variable investigation method (27) or a more advanced method like RSM to detect and finally optimize an integrated method to achieve high yield from extraction.

We used RSM only to correlate the values of independent factors, as randomly connecting independent values often leads to a mix-up of the values, thereby overlapping the independent factors. For an unbiased comparison between Soxhlet and UAE techniques, we applied the same values of parameters. The final values selected for subsequent experiments were 20 mL/g, 140 min, and 40°C.

#### 3.1.2. Comparison between Soxhlet and UAE methods

**Figure 1:**
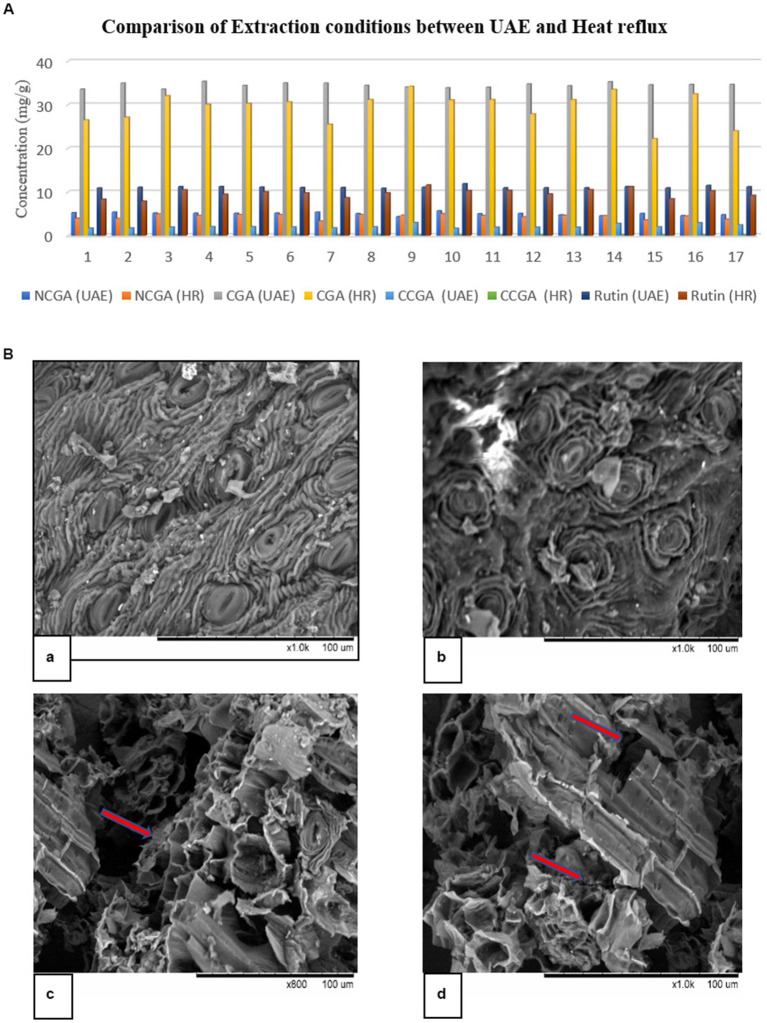
**(A)** Comparison of UAE and heat reflux using RSM. HR represents heat reflux. **(B)** Scanning electron microscopy (SEM) of DM after extraction: **(a)** and **(b)** Soxhlet; **(c)** and **(d)** UAE. The marked arrows present the cavitation and breaking phenomenon after sonication.

The two approaches, Soxhlet and UAE, were analyzed to determine which of them provides relatively high extraction of polyphenols (NCGA, CGA, CCGA, and rutin). The results are shown in Figure 1A. RSM generated 17 experimental runs (Supplementary Table S1), and all of the factors converged to provide the best extraction result. UAE yielded a high amount of the four polyphenols. Zhao et al. (28) extracted flavonoids from *Mitragyna speciosa*, and Peng et al. (29) isolated polyphenols from Chinese propolis using UAE, microwave-assisted extraction, and Soxhlet extraction. UAE gave a high yield of bioactive compounds compared to the other two methods.

A possible explanation for obtaining high extraction yields may be efficient disruption of plant tissues in UAE, which was supported by the SEM pictures (Figure 1B). SEM was used to observe any structural modifications during extraction for a better understanding of the two methods. Owing to the cavitation phenomenon, UAE treatment disrupted plant tissues and generated several hollow openings. Furthermore, these images revealed that ultrasound caused several cracks and openings, which allowed the efficient removal of compounds and saturation in the solvent.

### 3.2. Chemotactic analysis for discrimination and determination of polyphenols

#### 3.2.1. Bioactive compounds in different regions of the Republic of Korea

**Figure 2:**
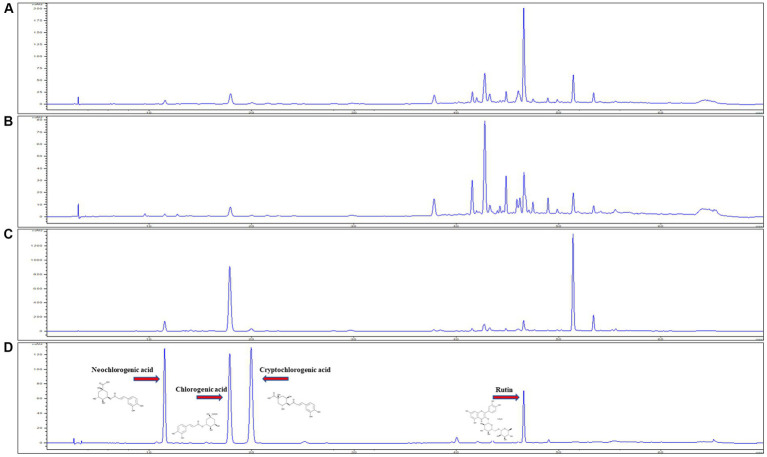
HPLC chromatogram of plant samples from three regions, **(A)** DMJ, **(B)** DMB, and **(C)** DMJG. **(D)** Analytical standards of the four compounds.

The developed method was used to simultaneously determine four polyphenols (CGA, NCGA, CCGA, and rutin) in three regions (DMJ, DMB, and DMJG) of the Republic of Korea (Figure 2). The lower LOD and lower LOQ were subsequentially applied to four compounds. In the DMJG, DMB, and DMJ regions, the four compounds quantified values were higher than LOD and LOQ. Out of the four bioactive compounds, CGA was highest within the DMJG region. Rutin was second highest, followed by NCGA and CCGA (Table 1). The pattern of quantified metabolites may be described as CGA > rutin > NCGA > CCGA based on their contents. In DM, quantification of bioactive molecules is going on, and a comparison among these four compounds has been provided by the present study. DM is widely used as a medicinal herb in regions of the Republic of Korea. Therefore, such therapeutic plant species needs to be evaluated in-depth in future studies to explain the reasons behind fluctuations in metabolites of DM plants in different regions.

**Table 1:** The concentration of four polyphenols in DM.

Contents of NCGA, CGA, CCGA and rutin (mg/g, *n* = 3)				
Batch No.	NCGA	CGA	CCGA	Rutin
DMJG 1	2.27 ± 0.09	16.19 ± 0.77	0.67 ± 0.04	4.81 ± 0.34
DMJG 2	2.39 ± 0.21	16.89 ± 1.65	0.68 ± 0.06	5.36 ± 0.45
DMJG 3	2.14 ± 0.05	10.7 ± 0.21	0.61 ± 0.03	4.48 ± 0.21
DMJG 4	2.51 ± 0.06	18.07 ± 0.48	0.97 ± 0.02	6.53 ± 0.41
DMJG 5	3.45 ± 1.21	22.63 ± 0.49	0.74 ± 0.04	6.79 ± 1.63
DMJG 6	3.62 ± 1.00	24.43 ± 0.23	1.01 ± 0.02	7.62 ± 1.02
DMJG 7	2.41 ± 0.03	17.89 ± 0.33	0.86 ± 0.03	6.25 ± 0.01
DMJG 8	3.42 ± 1.11	22.89 ± 0.57	0.79 ± 0.04	6.89 ± 1.35
DMJG 9	2.11 ± 0.04	14.47 ± 0.40	0.7 ± 0.04	4.47 ± 0.12
DMJG 10	2.25 ± 0.12	16.01 ± 0.16	0.77 ± 0.07	5.68 ± 0.33
DMJ 1	0.22 ± 0.01	0.37 ± 0.03	0.05 ± 0.03	6.89 ± 0.09
DMJ 2	0.19 ± 0	0.29 ± 0.01	0.03 ± 0.04	4.22 ± 0.10
DMJ 3	0.23 ± 0	0.49 ± 0.02	0.04 ± 0.02	4.58 ± 0.09
DMJ 4	0.22 ± 0.01	0.47 ± 0.04	0.06 ± 0.02	5.11 ± 0.85
DMJ 5	0.23 ± 0.01	0.33 ± 0.05	0.07 ± 0.02	5.59 ± 0.38
DMJ 6	0.23 ± 0.01	0.32 ± 0.02	0.07 ± 0.01	5.39 ± 0.14
DMJ 7	0.27 ± 0.01	0.47 ± 0.02	0.09 ± 0.02	6.58 ± 0.20
DMJ 8	0.26 ± 0	0.39 ± 0	0.09 ± 0.02	6.66 ± 0.22
DMJ 9	0.26 ± 0	0.41 ± 0	0.09 ± 0.03	6.45 ± 0.09
DMJ 10	0.24 ± 0.12	0.32 ± 0.01	0.08 ± 0.03	6.01 ± 0.18
DMB 1	0.12 ± 0	0.09 ± 0	0.07 ± 0.01	1.55 ± 0.09
DMB 2	0.12 ± 0	0.09 ± 0.02	0.07 ± 0.01	1.71 ± 0.05
DMB 3	0.13 ± 0	0.19 ± 0.02	0.08 ± 0	2.59 ± 0.78
DMB 4	0.12 ± 0	0.17 ± 0.02	0.07 ± 0.02	1.99 ± 0.03
DMB 5	0.13 ± 0	0.18 ± 0.02	0.08 ± 0	2.03 ± 0.04
DMB 6	0.14 ± 0	0.26 ± 0.03	0.08 ± 0	3.04 ± 0.03
DMB 7	0.13 ± 0	0.18 ± 0.01	0.08 ± 0	1.86 ± 0.03
DMB 8	0.13 ± 0	0.18 ± 0	0.08 ± 0	2.04 ± 0.07
DMB 9	0.12 ± 0	0.1 ± 0.03	0.07 ± 0.01	1.66 ± 0.49
DMB 10	0.13 ± 0	0.16 ± 0.01	0.09 ± 0.01	2.09 ± 0.06

^*^NCGA, neochlorogenic acid; CGA, chlorogenic acid; CCGA, cryptochlorogenic acid. DMJG: Jangheung region samples; DMJ: Jeju Island samples; DMB: Bogildo region samples.

#### 3.2.2. Multivariate statistical analysis

HCA is a clustering method that provides information on large amounts of data by organizing them into groups and subgroups depicting a hierarchy. In our experiment, HCA was carried out using Ward’s method as the cluster method. DM samples from the three regions were successfully classified into three groups, i.e., DMJG, DMJ, and DMB, according to the ratio of compounds. This indicates that the samples from different regions are undoubtedly different in terms of their polyphenol contents (Supplementary Figure S2).

**Figure 3:**
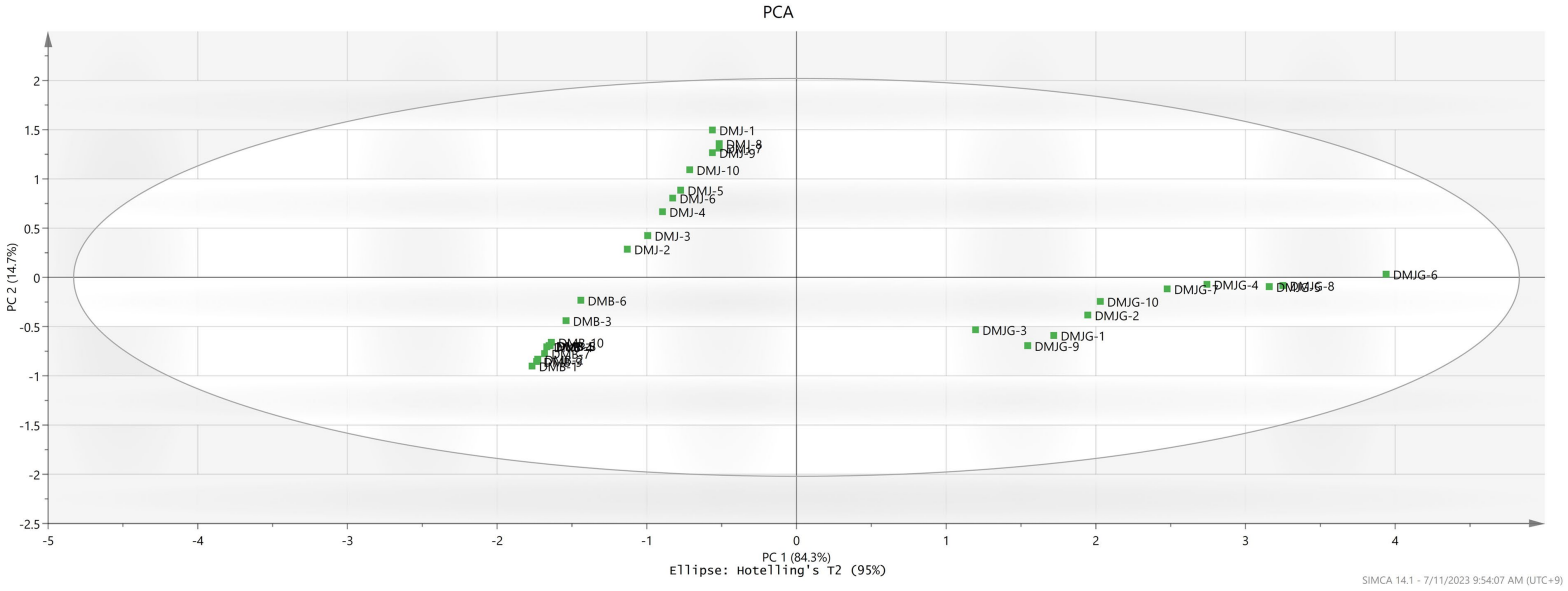
Representation of PCA in the three regions DMJ, DMB, and DMJG. The DMJG region has been plotted on the left side depicting the discriminative power of PSMs identified.

PCA and PLS-DA are mathematical approaches that can be useful to biological or chemical data to identify a pattern and classify them. Both methods are frequently used to discriminate and compare herbal medicinal plants (28). To provide a better understanding of the chemical compounds and potential marker metabolites in DM, both PCA and PLS-DA were carried out. Unsupervised PCA was performed to visualize and discriminate plants from the three regions based on quantification of the compounds. The first and second component analyses describe 84.3% and 14.7%, respectively (Figure 3). The plant materials were distinctly divided on the scatter plot, which was aligned with the HCA dendrogram (Supplementary Figure S2), where three different groups were made in the three regions (DMJ, DMB, and DMJG). Furthermore, DMJ and DMB were very close in the scatter plot, depicting almost the same amount of bioactive compounds. In contrast, the DMJG region was far from the other two, clearly indicating relatively high discrimination, which was aligned with the HCA plot where DMJ and DMB shared the same group of the hierarchy. In contrast, samples of the DMJG region were in a separate group. Furthermore, compared to DM PSMs in the three regions, the distribution of plant samples was scattered in DMJ and DMJG (Figure 3), which suggests that the quality of DM plant samples was less stable.

**Figure 4:**
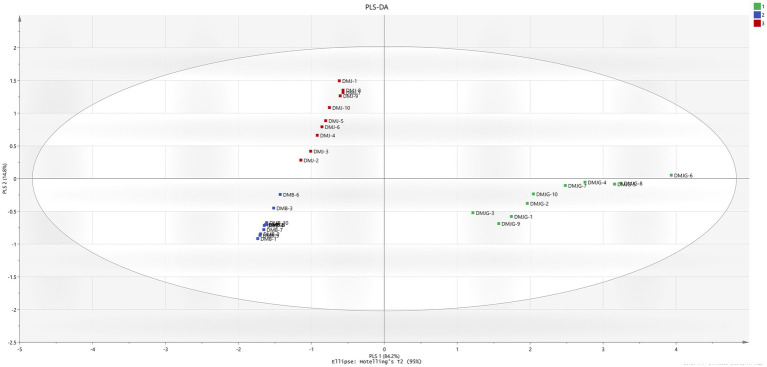
Representation of PLS-DA in the three regions, (1) DMJ, (2) DMB, and (3) DMJG.

To find potential markers in DMJ, DMB, and DMJG, PLS-DA was applied based on the quantities of the four compounds. Like the PCA result, the supervised PLS-DA score plot classified the samples into three groups with *R*^2^*Y* = 0.89 and *Q*^2^ = 0.882 (Figure 4). Furthermore, the variable importance in projection values were calculated, which represent the differences in variables and compounds that play important roles in differentiation, and values of PSMs >1.0 were picked out. Of the four compounds, the value for rutin only was 1.38863 (Supplementary Figure S3). It was selected as a responsible marker compound and played a significant role in intergroup differences in DM samples of the three regions. HCA, PCA, and PLS-DA have previously been used to discriminate plant species using metabolomics. Kwon et al. (30) distinguished ginseng leaves and varieties, and Chen et al. (31) performed similar work as ours on *Ganoderma lucidum* and used PCA, PLS-DA, and HCA to discriminate plant samples from different regions of China.

#### 3.2.3. Method validation of HPLC analysis

The developed method was validated using standard validation methods including linearity, LOD, LOQ, stability, precision, repeatability, and recovery. The acceptable linear correlation was set as (*r*^2^ ≥ 0.999). The LOD and LOQ of CCGA were 0.004 and 0.012 mg/mL, respectively, which were lowest. The relative standard deviations for inter-day and intraday precision were <1.937% and 1.636%, respectively. The stability was presented as RSD and was <1.827%. Furthermore, the repeatability of the four compounds was in the range between 0.584%–1.99%. The details of the method validation are provided in Table 2.

**Table 2:** The regression equation, *r*^2^, linear range, limit of quantification (LOQ), limit of detection (LOD), precision, stability, and repeatability of six analytes.

Compound	Regression equation	*r* ^2^	Linear range (mg/mL)	LOQ (mg/mL)	LOD (mg/mL)	Precision (RSD, %)		Stability (48 h) RSD (%)	Repeatability (*n* = 6) RSD (%)
						Intra-day (*n* = 6)	Inter-day (*n* = 9)		
NCGA	*y* = 14.374*x* − 64.217	0.999	0.003–1	0.026	0.008	0.936	0.918	1.216	0.584
CGA	*y* = 16.277*x* + 68.864	0.999	0.007–1	0.035	0.011	0.706	1.39	0.663	0.874
CCGA	*y* = 18.577*x* + 42.972	0.999	0.0009–1	0.012	0.004	1.135	1.937	1.656	1.99
Rutin	*y* = 5431.1*x* + 47.642	0.999	0.062–1	0.075	0.024	1.636	1.413	1.827	1.408

### 3.3. Discrimination of DM using single nucleotide polymorphism

#### 3.3.1. Analyzing genes in chloroplast genome to distinguish DM

DNA-based methods are effective against fraudulent practices. In agricultural, food, and plant-based sources, they are essential for valuable assessment, labeling, and authentication of medicinal plants. In our study, we selected three genes, two from chloroplast genome (cpDNA) that is has the least evolution rate and one (ITS2) from ribosomal region (rDNA) that is considered one of the best regions for DNA barcoding (29). In DM, until now, no work on discrimination using SNP has been performed. In the current study, distinguishing DM in the DMJG region was performed using SNP, which is reliable and easy (30). For discrimination DM, two genes, *petD*, and *trnL-trnF* in cpDNA were analyzed and screened to identify a stable SNP. Upon multi-alignment, in *petD*, no SNP was detected, whereas *trnL-trnF* had SNPs within the range of 911–970 bp. However, the SNPs were not very stable for designing a specific primer. The multi-aligned sequence, gel-based picture, and primer conditions for *trnL-trnF* and *petD* are provided in Supplementary Figures S4, S5, respectively.

#### 3.3.2. Authentication of DM in the DMJG based on ITS2 using multiplex PCR

**Figure 5:**
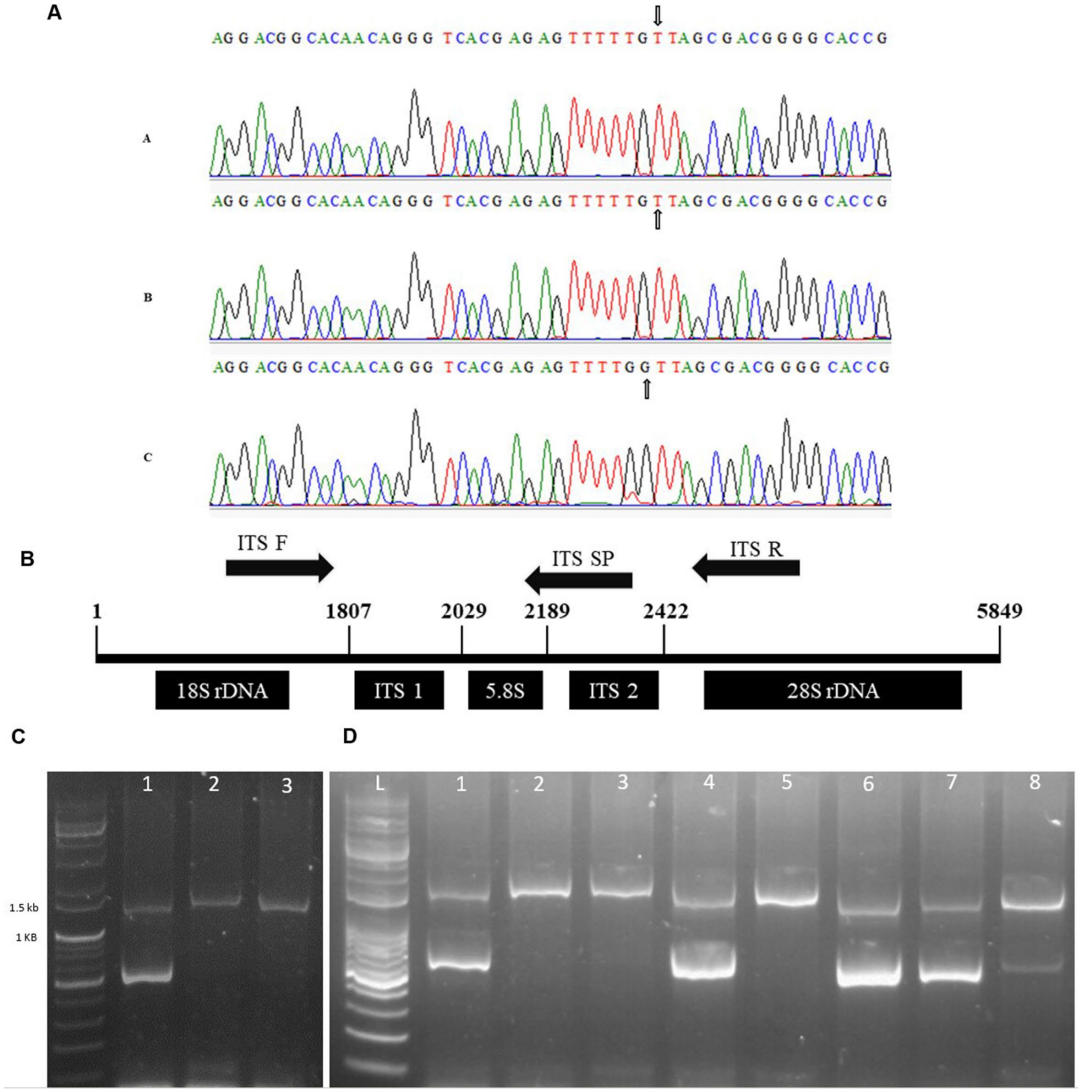
**(A)** Representation of SNP in the three regions, **(A)** DMJ, **(B)** DMB, and **(C)** DMJG. Nucleotide G in DMJG is replaced with T in the other two regions. The SNP site is represented with an arrow. **(B)** Primer set (forward and reverse) and a specific primer. The sequence is the actual presentation of 45S nrDNA sequence of DM, obtained from gene bank number “KT380923.1”. **(C)** Agarose gel image of the products of multiplex PCR, lane L, 1 kb DNA ladder; lane 2, DMJG; lane 3, DMJ; lane 4, DMB. **(D)** Lane L, 1 kb DNA ladder; lane 2, DMJG; lane 3, unknown sample; lane 4, unknown sample; lane 5, DMJG; lane 6, unknown sample; lane 7, DMJG; lane 8, DMJG; lane 9, DMJG.

After analyzing the two genes in cpDNA, we switched to rDNA. The ITS2 region was sequenced, and a stable SNP was detected upon multi-alignment in DMJG, where a nucleotide G was substituted with T. The SNP region is illustrated in Figure 5A, and the whole ITS sequences of plant samples from the three regions are provided in Supplementary Figure S6. The forward primer was used according to White et al. (31), and the reverse primer was designed downstream of the ITS2 region. A specific primer was designed for the SNP site in the ITS2 region (Figure 5B). To increase the specificity, an intentional mismatch C was introduced on the 3′ end of the primer (Table 3). DM in the DMJG region was authenticated using multiplex PCR, which yielded 1,400 bp products for samples from the three regions. However, applying the third primer specific to DMJG yielded a specific gel-based band of 581 bp (Figure 5C).

**Table 3:** Details of primers, sequence, ratio, melting temperature, and GC content used in multiplex PCR.

No.	Primers	Sequence	*T*_m_ (°C) and GC (%)	Primer ratio (μM)
1	ITS-F	GGAAGTAAAAGTCGTAACAAGG	51.3 and 40.9	1
2	ITS-R	ACCCTTCTCAGAAGATCAAG	51.3 and 45	1
3	ITS-R (SP)	AGGGTCACGAGAGT**C**TTG	53.6 and 55.6	0.6

Bold and underlined letters represent an intentional mismatch. F, R and SP represents the forward, reverse and specific primer respectively.

To analyze the efficiency and reproducibility of specific primers, DM plant samples were purchased online to perform a blind test. The results revealed the accuracy and reproducibility to be 100%. The test was carried out using eight samples, of which five belonged to the DMJG region, which was confirmed by the specific band of 581 bp and a common gel-based band of 1,400 bp (Figure 5D). Herein, we provided gel-based results to show the efficacy in discriminating DM samples in the DMJG region. Moreover, real-time PCR is also effective while authenticating plant species. Further research must be carried out to discriminate DM samples using real-time PCR.

### 3.4. *In silico* experiment

#### 3.4.1. Molecular docking and ADMET analysis

**Figure 6:**
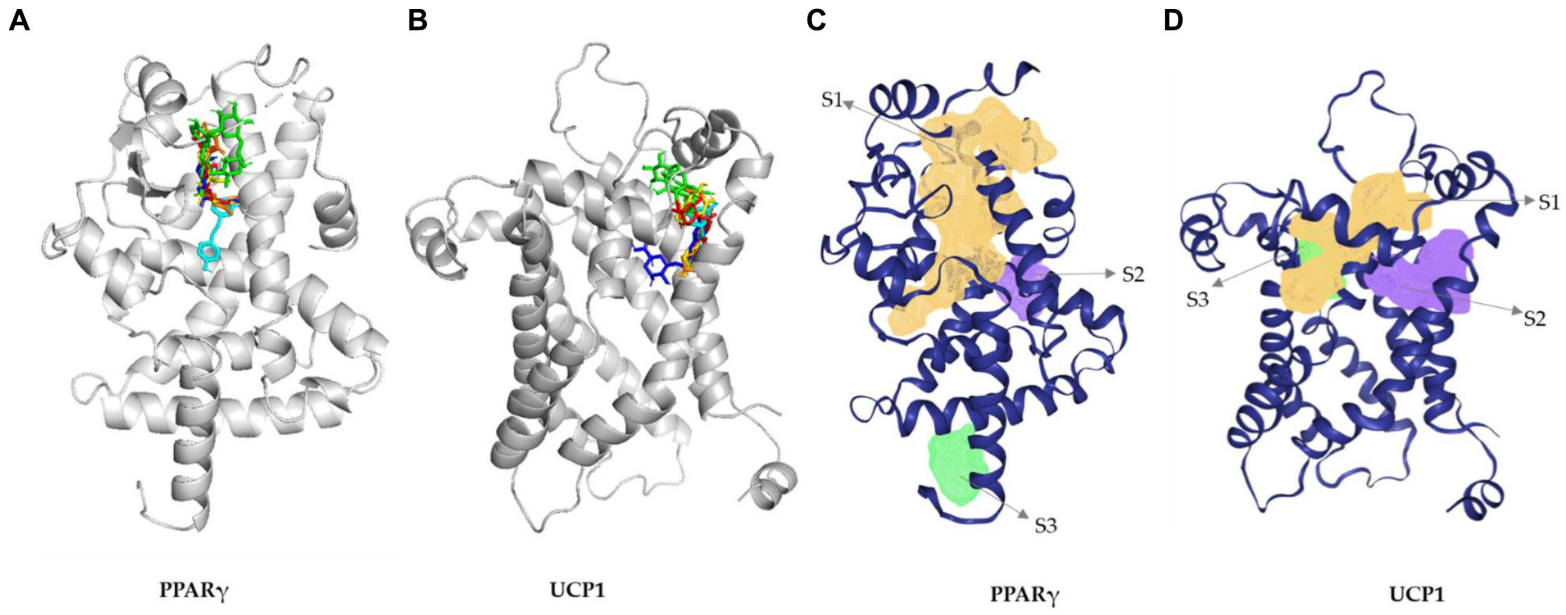
3D interaction diagram of PPAR*γ* **(A)** and UCP1 **(B)** with **(a)** rutin (green), **(b)** chlorogenic acid (blue), **(b)** neochlorogenic acid (yellow), **(c)** cryptochlorogenic acid (red), **(d)** resveratrol (cyan), and **(e)** rosiglitazone (orange). **(B)** Predicted active site for UPC1 gamma. **(B)** Predicted active site for PPAR*γ* **(C)** and UCP1 **(D)**.

Molecular docking is a widely used method in computer-aided drug design to identify potential drugs for various diseases. DM has been previously reported for its efficacy on various metabolic disorders (32, 33). In this present study, we screened four major phytochemicals (NCGA, CGA, CCGA, and rutin) from DM against two anti-obesity-related targets, PPARγ and UCP1. PPARγ is a master regulator of adipogenesis (34) and involved in various metabolic disorders. UCP1 is the best-characterized thermogenic effector and a key regulator of thermogenesis (35). The synergic effect of both PPARγ and UCP1 is crucial in managing obesity and related diseases. Therefore, we used rosiglitazone (36) and resveratrol (37) as control drugs. The docking results demonstrated that Rutin, CCGA, NCGA, and CGA used in this study against PPARγ had high binding energies of −8.2, −7.2, −7.3, and −7.7 kcal/mol, respectively. Rutin and CGA showed higher binding affinity than did the positive controls resveratrol and rosiglitazone having binding energies of −6.9 and −7.5 kcal/mol. Rutin and CGA formed four hydrogen bonds with GLU259, CYS285, SER342, ILE262, TYR327, SER289, CYS285, and SER342 residues. The amino acid residues TYR473, HIS449, SER289, and HIS323 have been previously reported to be crucial in inhibiting PPARγ. Interestingly, CGA, NCGA, and CCGA formed hydrogen bond with a reported amino acid residues (SER289) in the active site of PPARγ (Supplementary Table S4). The amino acid residues and binding pockets of the phytochemicals in DM were similar as those of the control drugs used in this study (Figure 6). Notably, the binding pockets of the phytochemicals were same as predicted (Figure 6 and Supplementary Table S5).

**Figure 7:**
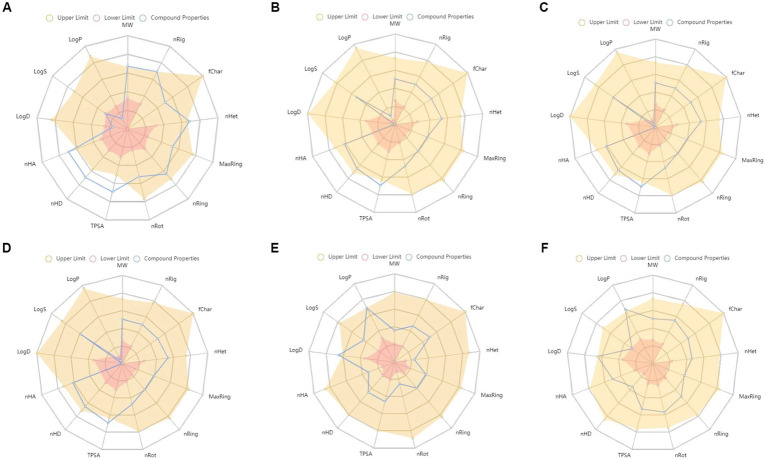
ADMET properties of **(A)** rutin, **(B)** cryptochlorogenic acid, **(C)** neochlorogenic acid, **(D)** chlorogenic acid, **(E)** resveratrol, and **(F)** rosiglitazone. MW, Molecular weight; nRig, number of rigid bonds; fChar, formal charge; nHet, number of heteroatoms; MaxRing, number of atoms in the biggest ring; nRing, number of rings; nRot, number of rotatable bonds; TPSA, topological polar surface area; nHD, number of hydrogen bond donors; nHA, number of hydrogen bond acceptor; LogD, logP at physiological pH 7.4; logS, log of the aqueous solubility; and LogP, log of the octanol/water partition coefficient.

Rutin, CCGA, NCGA, and CGA had significant binding energy of −8.4, −7.1, −7.2, and −6.8 kcal/mol for UCP1, respectively. Rutin showed the highest binding affinity among the compounds and drugs used in this study (Supplementary Table S6). Furthermore, the binding pockets and amino acid residues were the same as those of the control drugs and were similar to the predicted ones (Figures 6B,D and Supplementary Table S5). Rutin formed eight hydrogen bonds with the target at LYS73, ARG84, THR36, GLN144, HIS146, GLN142, LEU141, and LEU147 residues (Supplementary Table S6). The 2D structures, binding score, and details of interactions of all the screened compounds are displayed in Supplementary Tables S4−S6 and Supplementary Figures S7, S8. Finally, ADMET of all the compounds was predicted and analyzed (Figure 7 and Supplementary Tables S7−S12). As a result of these *in silico* studies, the compounds present in DM showed promising results. Therefore, DM could potentially be used to manage obesity and metabolic disorders by synergizing the activities of PPARγ and UCP1.

### 3.5. *In vitro* experiment

#### 3.5.1. Effect of DM extracts on cell viability

**Figure 8:**
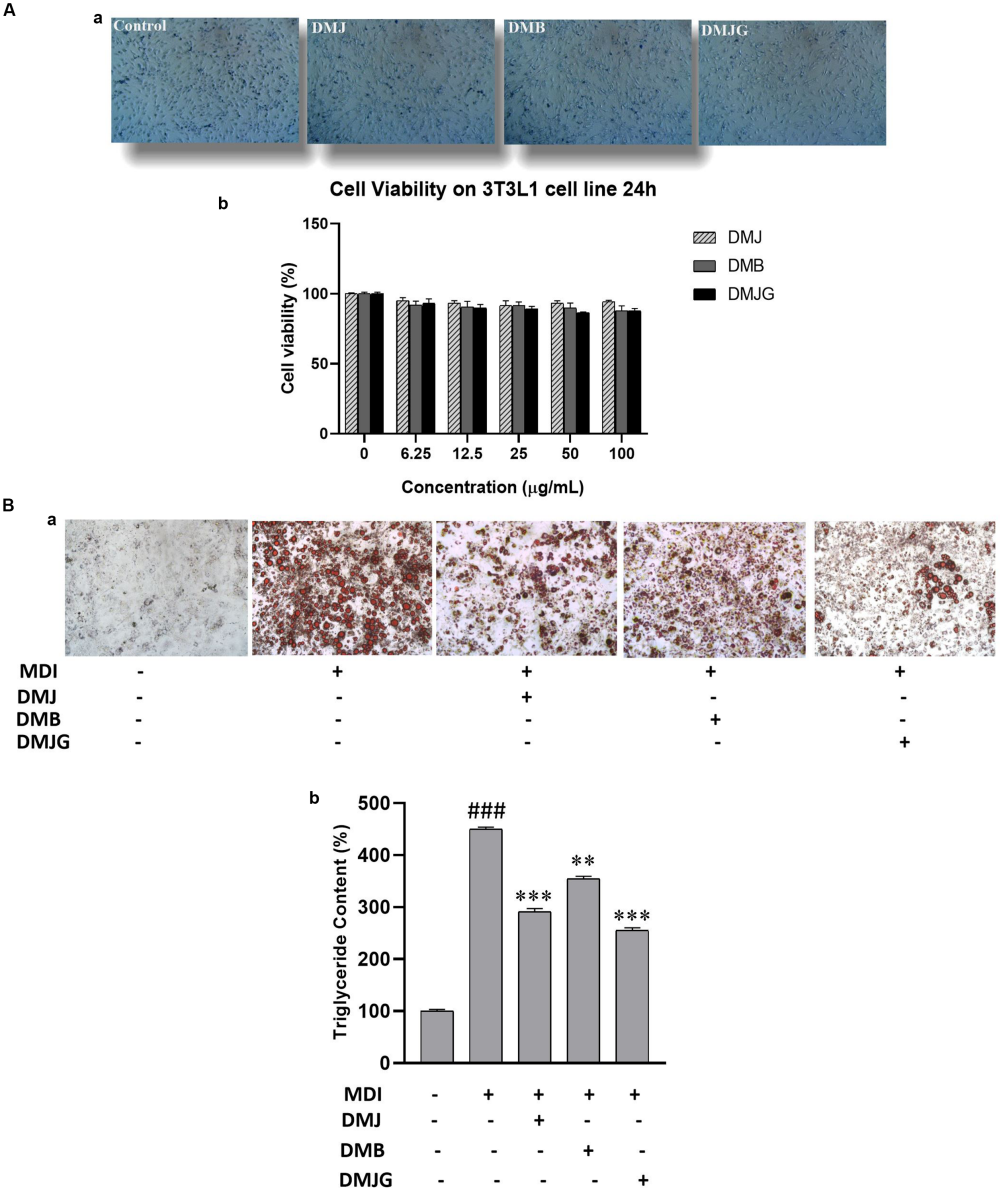
**(A)** Cell viability **(a)** effect of treatment of DM extracts on 3T3-L1 cells for 24 h. **(b)** Trypan blue assay before and after treatment. **(B)** Inhibition of lipid deposition by DM extract treatment on MDI-induced 3T3-L1 adipocytes. **(a)** Lipid droplets were visualized by oil red O staining using a fluorescence microscope. **(b)** Triglyceride content was measured after dissolving oil red O in isopropyl alcohol (520 nm). The data are mean values of three experiments ± standard error (SEM). ^###^<0.001 compared with control, ^**^p < 0.01, ^***^ *p* < 0.001 compared with the data of MDI treatment.

Adipose tissue stores excess energy in the form of triglycerides derived from food. Mouse 3T3-L1 preadipocyte cells were used as a cellular model because they present all adipocytes (38). We investigated the cell viability of different doses (6.25–200 μg/mL) of DM (DMJ, DMB, DMJG) using MTT assay. Results showed no significant changes in cell viability. Therefore, we selected 100 ug/mL DM dose for further experiments (Figures 8A,B).

#### 3.5.2. Inhibitory effects of DM on lipid accumulation

Adipocytes play important roles in lipid metabolism and energy homeostasis; however, an increase in their number in adipose tissues leads to obesity. Moreover, obesity is caused by adipogenesis and lipid buildup in adipocytes. Therefore, inhibiting lipid accumulation in adipocytes is a principal target for preventing obesity and related disorders (39). Fat droplets in adipocytes were visualized using oil red O (40). It has been observed that 3T3-L1 preadipocytes differentiate in a specified adipogenic solution. Thus, Adipocyte differentiation was initiated using MDI solution with DM extracts for 8 days, and anti-obesity activity was assessed using oil red O staining in differentiated 3T3-L1 cells. Microscopic observation indicated the number of lipids present in treated and nontreated cells. The initiation of differentiation *in vitro* results in hyperplasia (an increased number of adipocytes) and hypertrophy (an increased size of adipocytes) (41). Our result exhibited that DM extracts (DMJ, DMB, DMJG) reduced the number and size of lipid droplets (Figure 8B).

Our result was further confirmed by quantitative analysis of lipid accumulation. Cellular triglyceride content was notably increased in MDI-treated mature adipocytes during adipogenesis *in vitro.* However, when DM extract was added into the MDI medium, triglyceride content significantly reduced. DMJG (100 μg/mL) demonstrated a significant lipid-reducing effect via suppressing lipid accumulation by approximately 45%, and DMB (100 μg/mL) suppressed lipid accumulation by up to 20% (Figure 8C).

#### 3.5.3. Effect of DM on the expression of adipogenesis and thermogenesis-related genes

**Figure 9:**
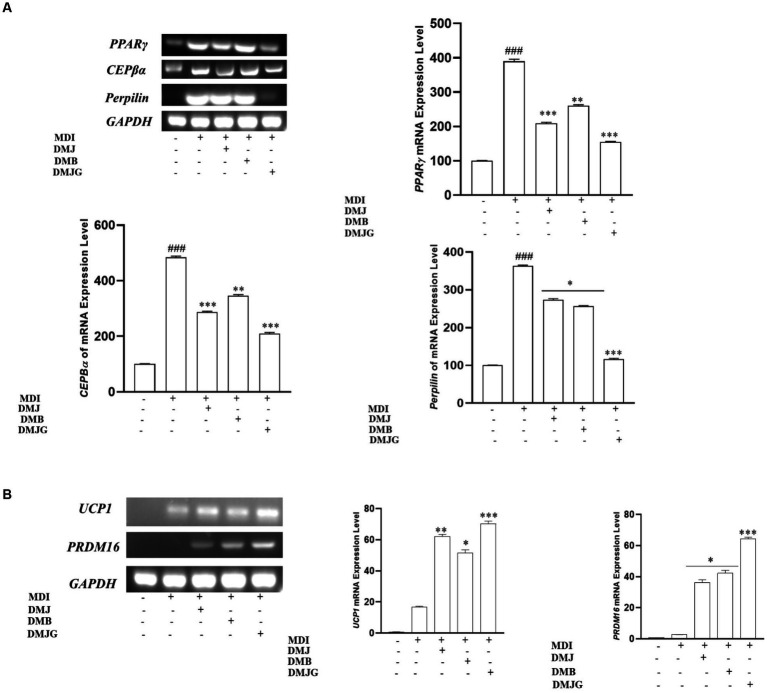
**(A)** Effect of DM extracts on mRNA expression of adipogenesis-related genes in MDI induced 3T3-L1 cells. DM extracts significantly inhibited the mRNA level of adipogenesis-related genes. ^###^<0.001 compared with control, ^*^p < 0.05, ^**^p < 0.01, ^***^p < 0.001 compared with the data of MDI treatment. **(B)** Effect of DM extracts on mRNA expression of thermogenesis-related genes in MDI-induced 3T3-L1 cells. DM extracts significantly upregulated the mRNA level of thermogenesis-relatedk genes. ^*^p < 0.05, ^**^p < 0.01, ^***^p < 0.001 compared with the data of MDI treatment.

Adipogenesis is the process through which adipocyte precursor cells develop into mature adipocytes. Preadipocytes must express several transcription factors, including as PPARγ, C/EBPα, and perilipin, in order to develop into mature adipocytes. Hence, we performed RT-PCR to find out if DM extracts can inhibit adipogenesis by inhibiting adipogenesis-specific transcription factors (PPAR*γ*, CEBPα, and perilipin). PPAR*γ*, CEPBα, and perilipin are major regulators that are closely related to lipid contents in adipocytes (42). Activation of *PPARγ* enhances *C/EBPα* activity, which accelerates pre-adipocyte differentiation to adipocytes and promotes adipogenesis and lipogenesis that result in increased body weight (43). Perilipin, located on the surface of differentiated 3T3-L1 adipocytes, is associated with promoting formation of lipid droplets and inhibiting lipolysis in adipocytes (44). These biomarkers play a separate but essential role in adipogenesis. MDI-treated 3T3-L1 cells activate the adipogenic transcription factors, including *CEBPα* and *PPARγ* (45). We observed that DM extracts selectively downregulated the mRNA expression of *CEBPα* along with *PPARγ* and perilipin expression (Figure 9A). More specifically, the DMJG plant extract suppressed gene expression of all these biomarkers better than DMJ and DMB plant extracts.

Moreover, research on boosting thermogenesis or browning has lately gained attraction since it causes weight loss through energy expenditure. Consequently, using recruitable brown adipocytes is an effective strategy for treating and preventing obesity. UCP1 and PRDM16 are two key biomarkers that play an essential role in regulating energy expenditure or thermogenesis (46). Thermogenesis is influenced by oxygen consumption by cells, and activation of UCP1 causes heat liberation by uncoupling the electron transport chain from energy production. PRDM16 activates thermogenesis-related transcription factors such as UCP1 (47). In the present study, DM extracts upregulated thermogenesis-related gene expression, and DMJG led to a significant increase in *UCP1* and *PRDM16* expression compared to that by DM of the other two regions (Figure 9B).

These results suggest that DM from the three different regions can inhibit obesity via downregulating adipogenic markers and upregulating thermogenic markers. DMJG showed relatively high activity against obesity, which indicates that the efficacy of cell lines is affected by PSMs, as DMJG offered the highest anti-obesity effects on adipogenesis and thermogenesis.

#### 3.5.4. Effect of DM on ROS in 3T3-L1 cells

In a normal body state, the production of ROS and their destruction by antioxidants are tightly regulated (48). In obese conditions, ROS increase the differentiation of preadipocyte to adipocyte (49). High amount of ROS is produced by mitochondrial dysfunction, and intracellular ROS generation causes excessive lipids accumulation, resulting in adipocyte differentiation (50). This phenomenon may cause several metabolic disorders, including cancer (51). Therefore, suppression of ROS production using natural resources with high antioxidant activity could be inevitable in developing new drugs with few side effects to treat obesity.

In MDI-treated cells, an increase in ROS generation was noticed, which was suppressed through treatment with DM extracts from all three regions. DMJG showed the highest efficacy in inhibiting ROS generation and suppressed approximately 50% ROS production in MDI-treated cells. Therefore, reduction in lipid growth and ROS generation indicates anti-adipogenesis and ROS-suppressive effects of DM extract (Supplementary Figure S9).

### 3.6. Total phenols and antioxidant activities

Total phenols in medicinal plants are considered as important bioactive compounds with antioxidative activities (52). In the present study, the levels of total phenols were compared among the plant samples of the three regions, which were in the range of 1.9–6.657 mg GAE/g (milligrams of gallic acid equivalent per gram dry weight of sample). DMJG had the highest content of total phenols, followed by those of DMJ and DMB (Table 4).

**Table 4:** Total phenolics, DPPH scavenging activity, and ABTS activity.

	Total phenolics (mg GAE/g)	DPPH scavenging mg AAE/g	DPPH scavenging IC50 (mg/mL)	ABTS	ABTS IC50 (mg/mL)
DMJG	6.657	10.625	0.781	9.197	0.855
DMJ	2.696	10.437	1.98	8.438	2.525
DMB	1.9	7.041	3.609	7.178	3.393

For assessing the antioxidative properties of DM, two frequently used methods, DPPH and ABTS, were performed, which are simple, robust, and reproducible for accessing the antioxidant activities of plant samples. Table 4 shows the antioxidant activities of DM samples from the three regions, and DM exhibited antioxidative properties. DMJG showed the highest antioxidative efficacy, followed by those of DMJ and DMB.

## 4. Conclusion and future perspectives

**Figure 10:**
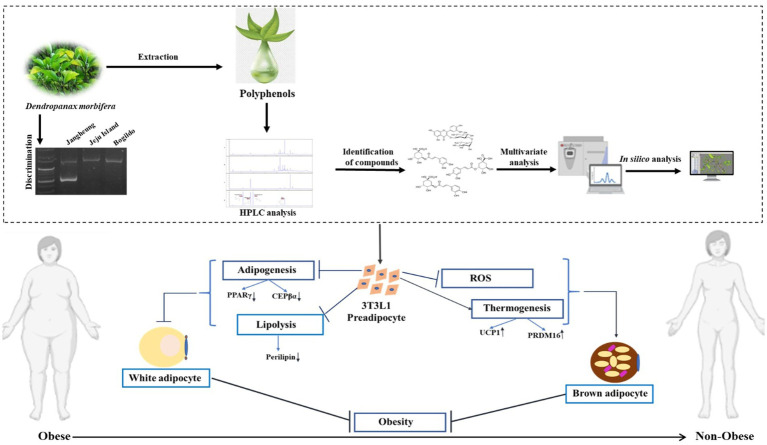
Schematic representation of the work performed in this article.

In conclusion, a detailed study was carried out on DM, which provided an understanding of plant samples from different regions of the Republic of Korea by understanding the regional variation of PSMs in plants followed by *in silico* analysis to identify the polyphenols that are responsible for downregulating major genes for adipogenesis and upregulating principal genes for thermogenesis. In addition, SNP analysis was performed for discriminating the plant samples in the DMJG region. A holistic scheme of the work is presented in Figure 10. The present work opens new pathways of understanding and carrying out studies on plants rich in secondary metabolites, taking into consideration the plant samples from different areas to search for samples that are enriched with high PSMs, to enhance their efficacy on animal cells and to distinguish them for preserving their effects on an industrial scale. To conclude, our current study addresses some core facts on DM. Moreover, we have performed some experiments (data not provided) on DM regarding age-related PSMs. Further experiments need to be conducted to explore how the age of DM plays an important role in obtaining high contents of PSMs.

## Data availability statement

The ITS sequences of DM can be found in the NCBI, DMJG (OR263193), DMJ (OR263482), and DMB (OR263483). The sequences for *petD* and *trnL-trnF* can be acquired upon reasonable request from MA (awaiskazmi@khu.ac.kr).

## Author contributions

MA contributed to the data collection and plant material experiment and wrote the first draft of the manuscript. RA performed experiments on cell work. VB carried out *in silico* analysis. JA helped with the HPLC analysis. JL helped with plant samples. Y-JK, RM, G-YK, MR, and GL edited the manuscript. MA, DY, Y-JK, and S-KJ contributed to the conception and design of the manuscript. All authors contributed to the article and approved the submitted version.

## Funding

This work was supported by Southwest Coast Hwangchil Cooperative.

## Conflict of interest

The authors declare that the research was conducted in the absence of any commercial or financial relationships that could be construed as a potential conflict of interest.

## Publisher’s note

All claims expressed in this article are solely those of the authors and do not necessarily represent those of their affiliated organizations, or those of the publisher, the editors and the reviewers. Any product that may be evaluated in this article, or claim that may be made by its manufacturer, is not guaranteed or endorsed by the publisher.

## Footnotes

^1^ foodsafetykorea.go.kr

^2^ http://multalin.toulouse.inra.fr/multalin/

^3^ https://www.idtdna.com/pages/tools/oligoanalyzer
