# Supplementary material for: Discrimination of Dendropanax morbifera via HPLC fingerprinting and SNP analysis and its impact on obesity by modulating adipogenesis- and thermogenesis-related genes
Source: Front Nutr. 2023 Aug 2;10:1168095. doi: 10.3389/fnut.2023.1168095 (PMC10446900; doi:10.3389/fnut.2023.1168095)
Supplement: Supplementary file 1 [file Presentation_1.pdf]

Discrimination of *Dendropanax morbifera* via HPLC fingerprinting and SNP analysis and its impact on obesity by modulating adipogenesis- and thermogenesis-related genes

Muhammad Awais[1], Reshmi Akter[1], Vinothini Boopathi[1], Jong Chan Ahn[1], Jung Hyeok Lee[1], Ramya Mathiyalagan[1], Gi-Young Kwak[1], Mamoona Rauf [3], Deok Chun Yang [1,2], Geun Sik Lee [4, 5], Yeon-Ju Kim [1]*, and Seok-Kyu Jung [6]*

[1]Graduate School of Biotechnology, College of Life Sciences, Kyung Hee University, Yongin si, 17104 Gyeonggido, Republic of Korea; awaiskazmi@khu.ac.kr; reshmiakterbph57@gmail.com; vinothiniboopathi@khu.ac.kr; jongchanahn7@khu.ac.kr; wndgur0144@gmail.com; ramyabinfo@gmail.com; kwakgiyoung8@gmail.com; dcyang@khu.ac.kr; yeonjukim@khu.ac.kr

[2]Department of Oriental Medicinal Biotechnology, College of Life Sciences, Kyung Hee University, Yongin-si, 17104 Gyeonggi-do,17104, Republic of Korea

[3]Department of Botany, Abdul Wali Khan University Mardan, 23200, Pakistan; mamoona@awkum.edu.pk

[4]Southwest Coast Hwangchil Cooperative, Chonnam National University, Yongbong-dong, Gwangju si, 61186 and Yeji Bio, #411, Korea; 1357oklee@naver.com

[5]Jungwon University Industry Academic Cooperation Building, Goesan-gun, Chungbuk 28024, Korea

[6]Department of Horticulture, Kongju National University, Yesan, 32439, Korea; jungsk@kongju.ac.kr

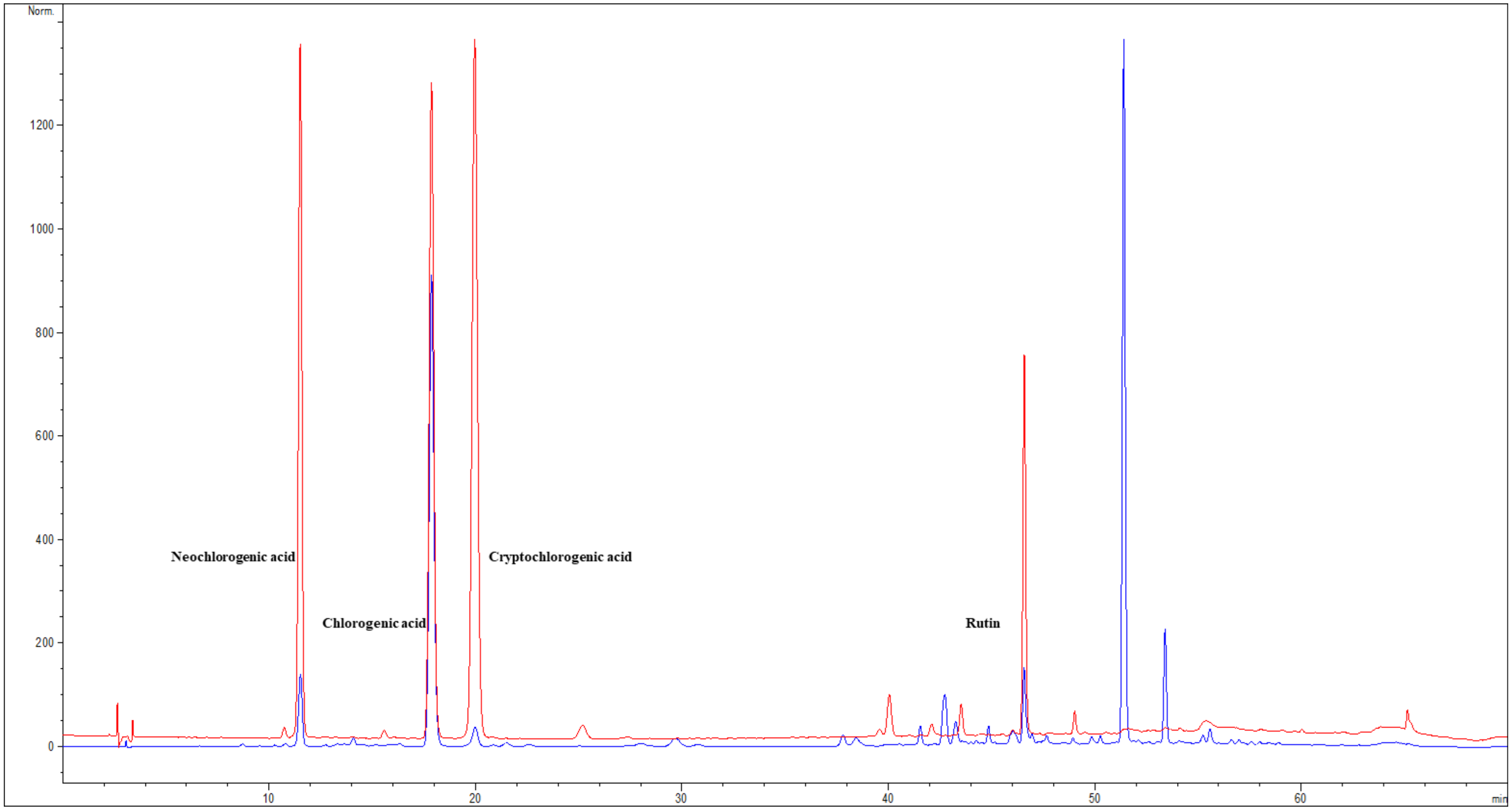

Figure S1 Merging the analytical standards and sample peaks to provide details in the same retention time. The Red line represents the analytical standards, while the blue line represents the DM sample.

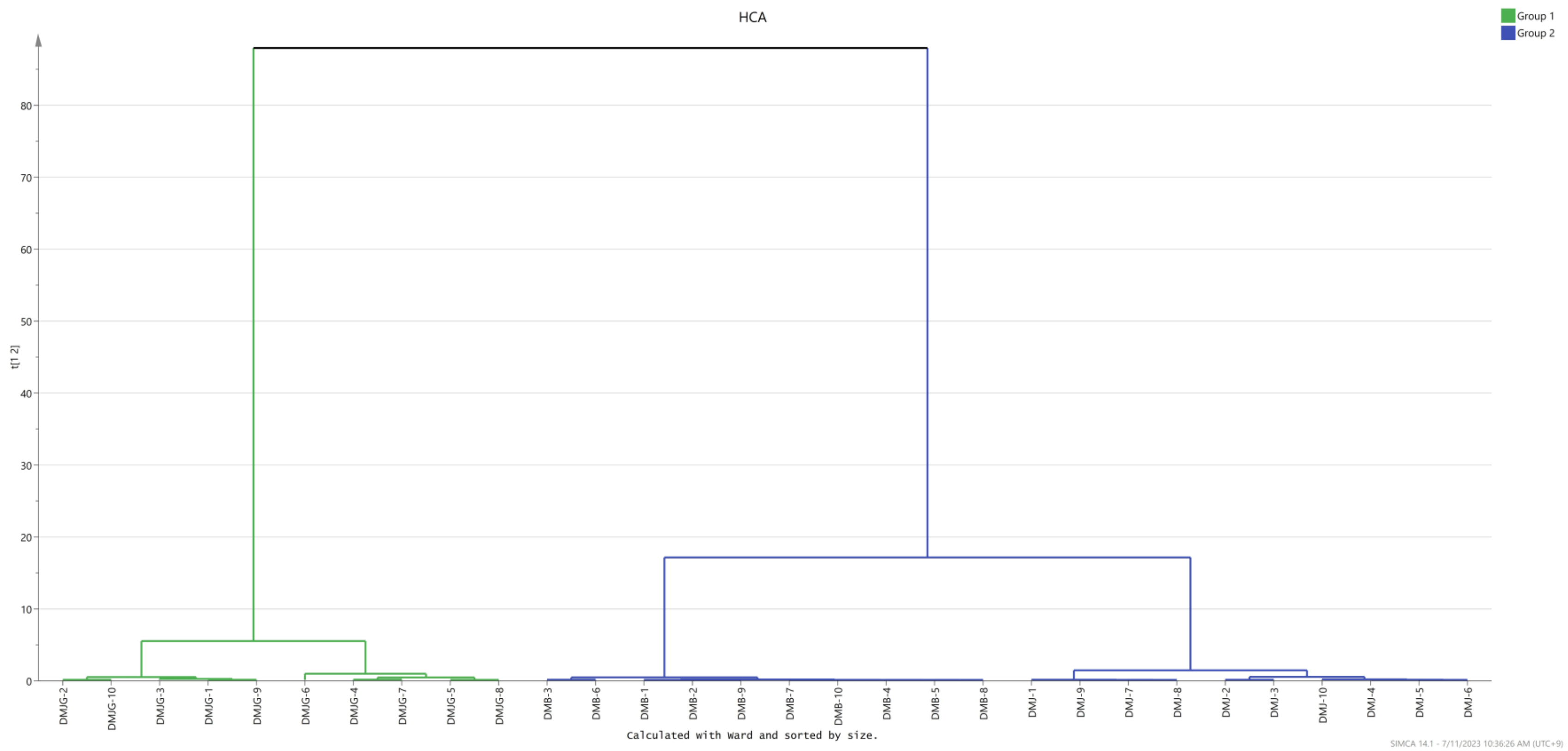

Figure S2 Representation of dendrogram in the three regions. The blue line represents one group of the two areas (DMB and DMJ), whereas the green line represents a separate lineage representing the DMJG region.

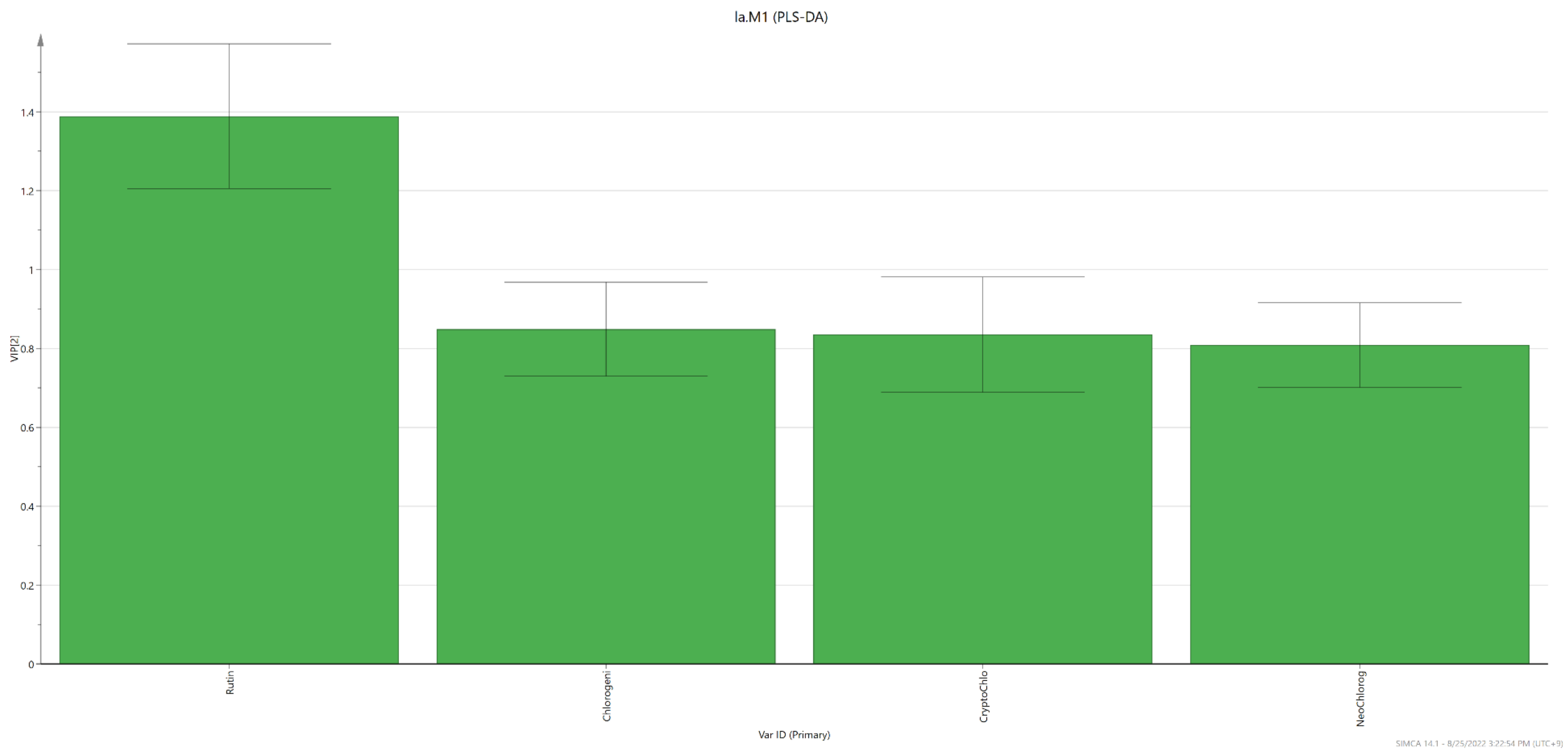

Figure S3 Representation of variable importance in projection VIP values. Only Rutin has a value > 1.

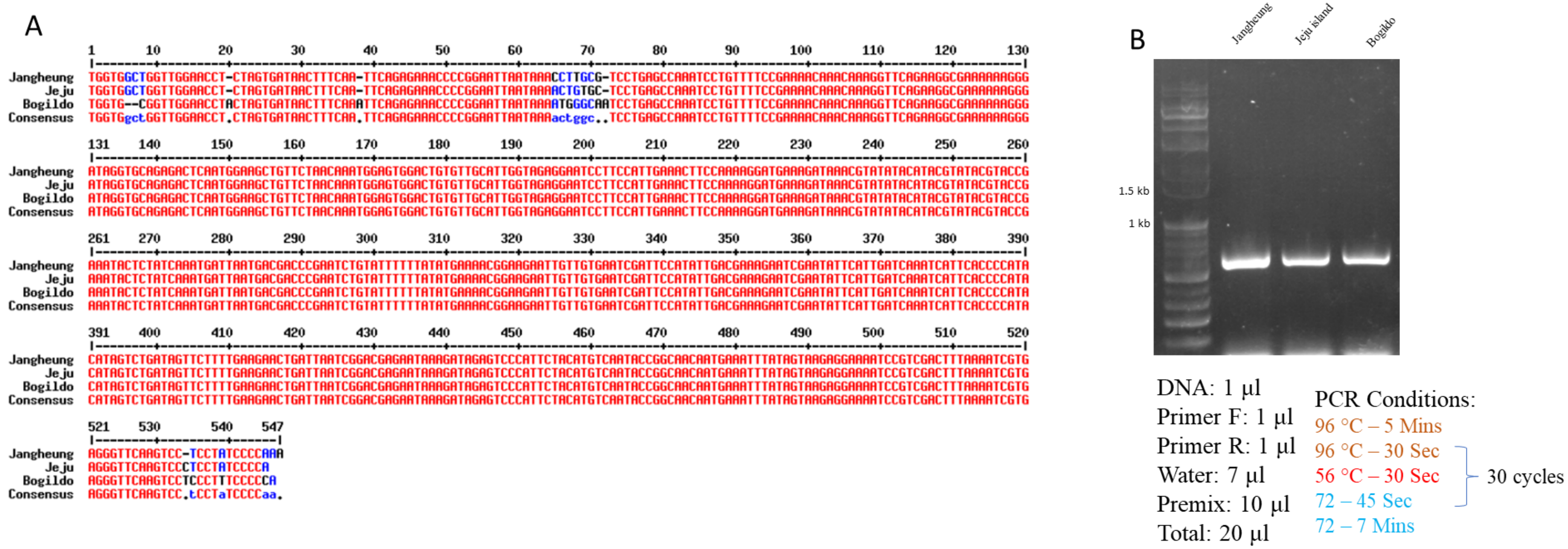

Figure S4 (A) Multialinged sequence of the *trnl-trnF* gene in the three regions, Jangheung, Bogildo, and Jeju island. (B) Gel-based picture and conditions for the polymerase chain reaction (PCR).

A

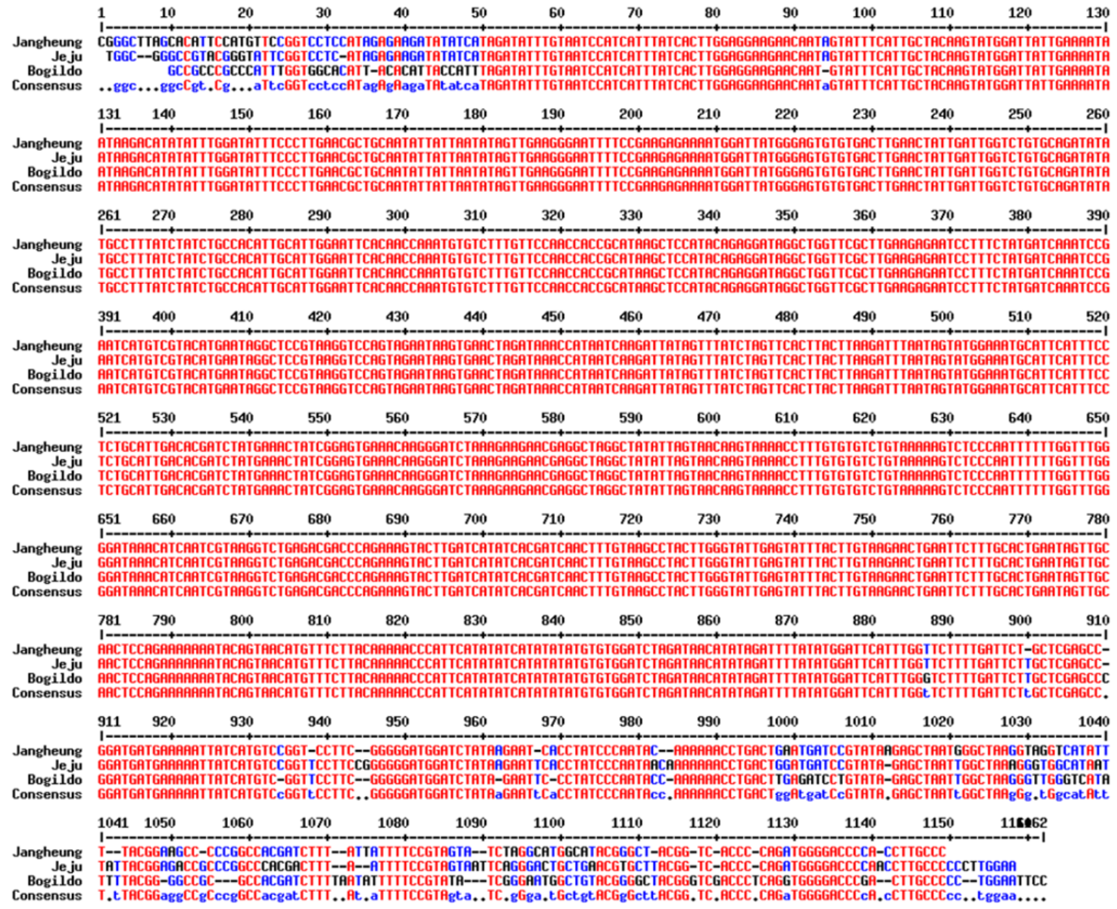

B

Forward primer

GGG AGT GTG TGA CTT GAA CTA TTG **GC: 45.8% Tm: 55.6 °C**

Reverse primer

GGG TAT TGG TGC AAC ATT ACC **GC: 48% Tm: 58.5 °C**

C

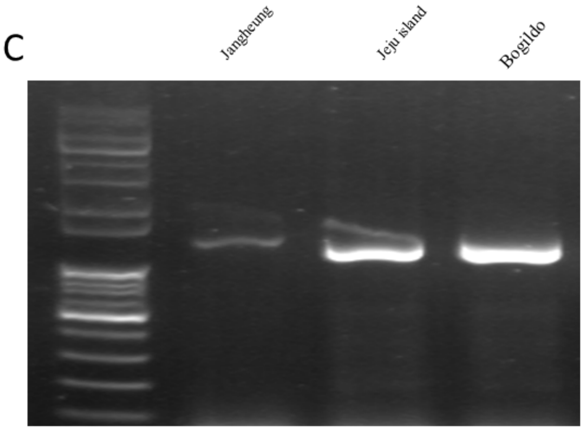

DNA: 1 µ l (ng/µl)
Primer F: 1 µl (10 pmol/ µl)
Primer R: 1 µl (10 pmol/ µl)
Water: 7 µl
Premix: 10 µl
Total: 20 µl

PCR Conditions:
96 °C – 4 Mins
96 °C – 30 Sec
58°C – 30 Sec } 36 cycles
72 – 1 min
72 – 7 Mins

Figure S5 (A) Multialinged sequence of the *petD* gene in the three regions, Jangheung, Bogildo, and Jeju island. (B) primer set used for the *petD* gene. (C) Gel-based picture and conditions for the polymerase chain reaction (PCR).

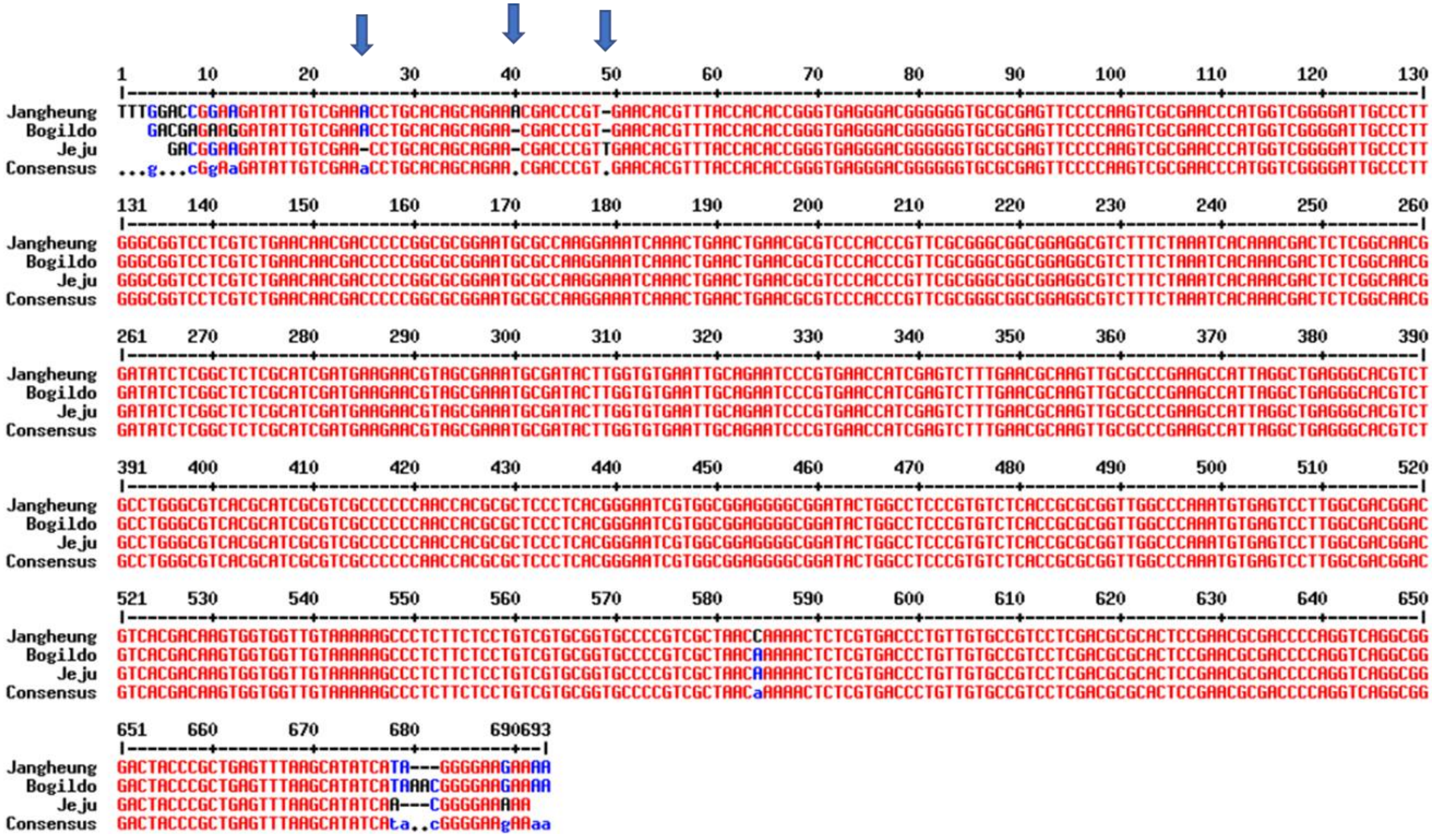

Figure S6 The multialinged sequence of the ITS region in the three regions is provided. The three blue arrows represent stable SNP regions.

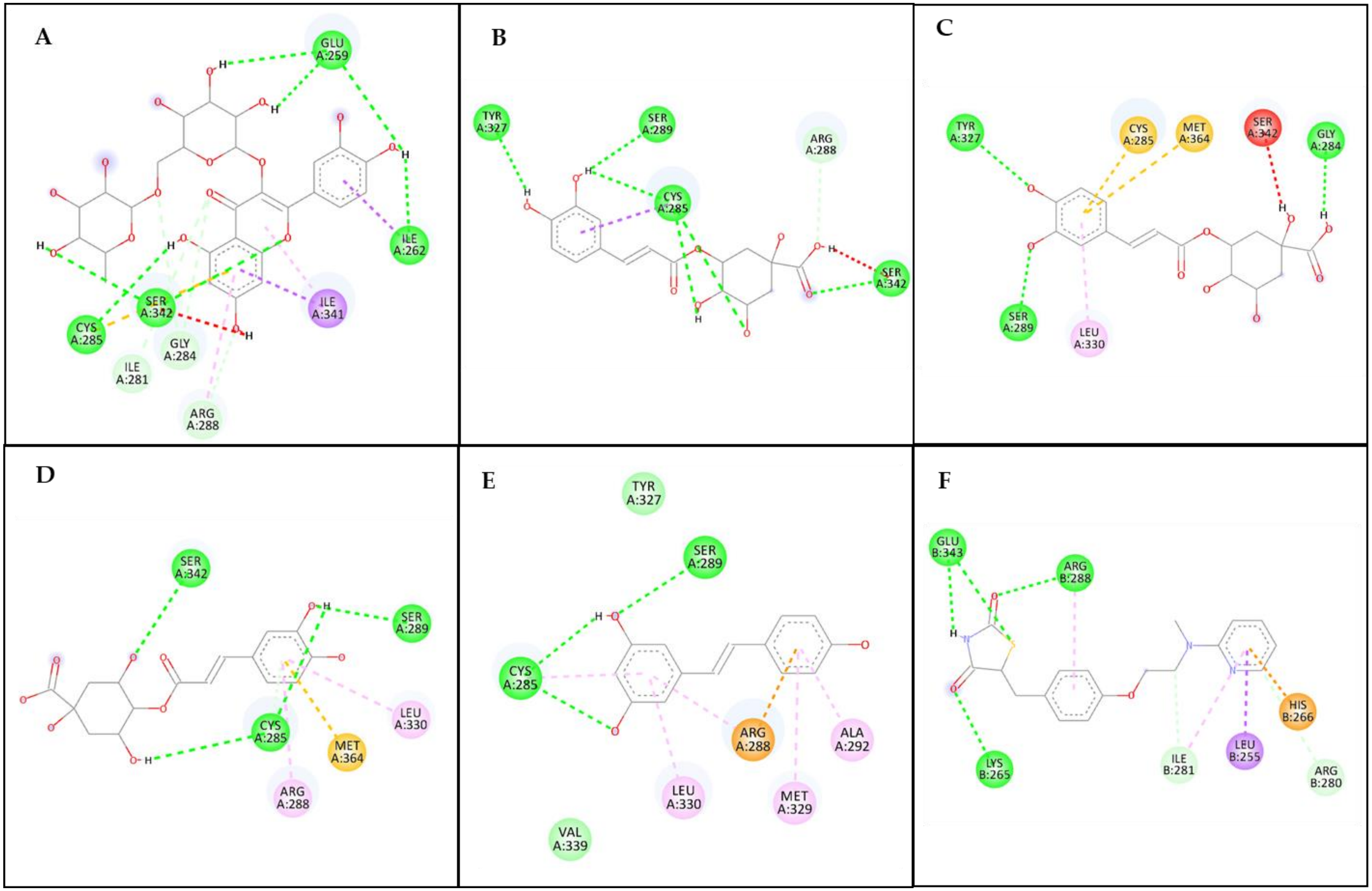

Figure S7 2D interaction diagram of PPAR gamma with (A) Rutin, (B) Chlorogenic acid, (C) Neochlorogenic acid, (D) Cryptochlorogenic acid, (E) Resveratrol, and (F) Rosiglitazone.

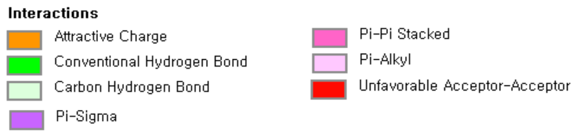

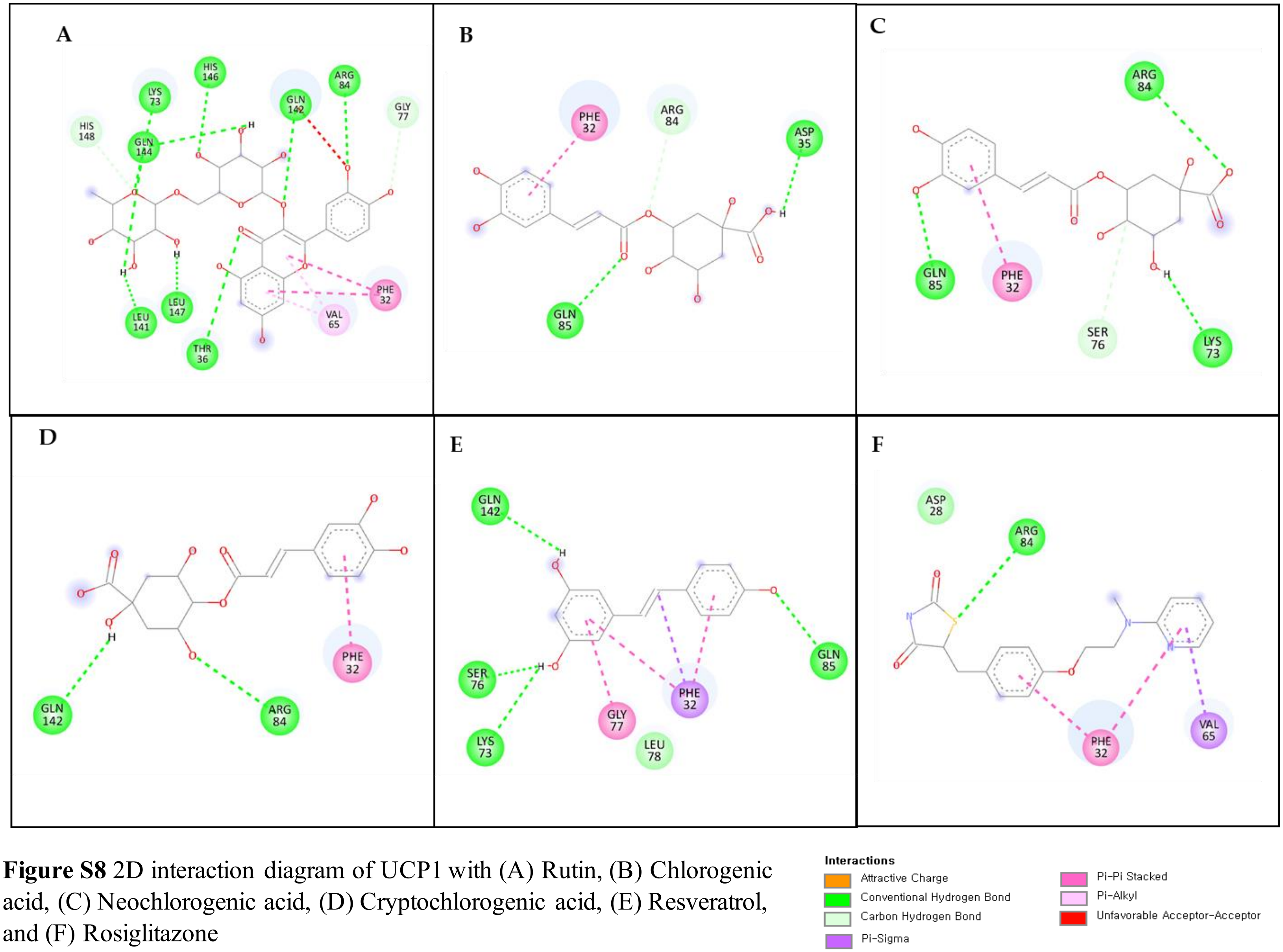

Figure S8 2D interaction diagram of UCP1 with (A) Rutin, (B) Chlorogenic acid, (C) Neochlorogenic acid, (D) Cryptochlorogenic acid, (E) Resveratrol, and (F) Rosiglitazone

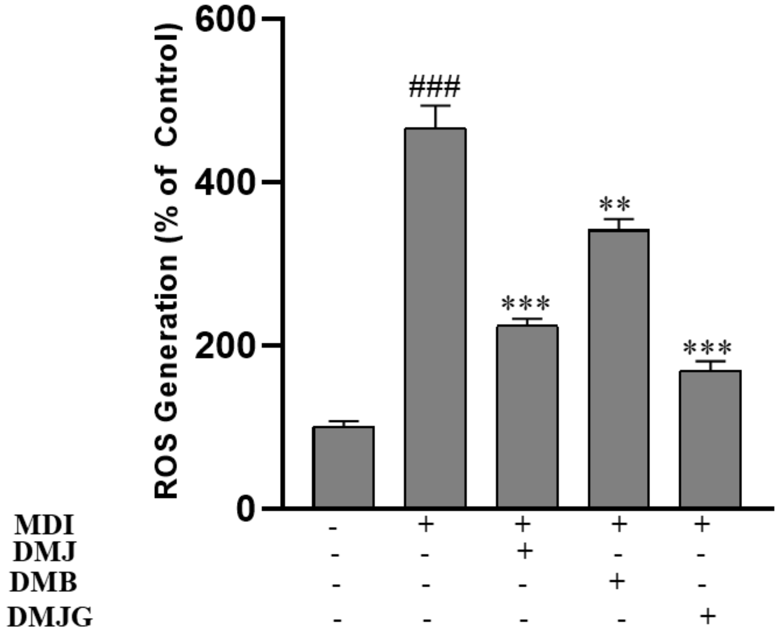

Figure S9. Inhibition of reactive oxygen species (ROS) generation by DM extracts in MDI-induced 3T3-L1 adipocytes was determined by DCFDA method. DM extracts reduced intracellular ROS production significantly. Data are expressed as a percentage of control. ### <0.001 compared with control, ** $p < 0.01$, *** $p < 0.001$ vs. MDI.

Table S1. RSM generated 17 experimental runs. The highlighted values are used in the manuscript for subsequent experiments.

Run	ml/gm	Min	Temperature
1	20	140	40
2	30	100	40
3	20	100	60
4	40	180	60
5	20	180	60
6	30	140	60
7	30	180	40
8	30	140	60
9	30	180	80
10	40	140	40
11	30	140	60
12	40	100	60
13	30	140	60
14	40	140	80
15	30	140	60
16	20	140	80
17	30	100	80

Table S2. Grid box coordinates and size parameters used for molecular docking**.**

	**PPAR gamma**	**UCP1**
**Dimension (Å)**		
x	90	66
y	124	92
z	94	72
**Centre (Å)**		
x	−21.85	−1.726
y	20.018	-7.316
z	-14.44	16.226

Table S3. Primers used for the RT-PCR are listed below.

No.	Primers	Sequence	Reference
1	PPARγ-F	ATGGGTGAAACTCTGGGAGATT	
	PPARγ-R	AGCTTCAATCGGATGGTTCTT	
2	C/EBPα-F	TTCATGGAGAATGGGGGCAC	(1)
	C/EBPα-R	TAGACGTGCACACTGCCATT	
3	GAPDH-F	GTATGACTCCACTCACGGCAAA	
	GAPDH-R	GGTGTGGCTCCTGGAAGATG	
4	perpilin-F	GATCGCCTCTGAACTGAAGG	(2)
	perpilin-R	CTTCTCGATGCTTCCCAGAG	
5	UCP1-F	TAAAAACAGAAGGGCGGATG	
	UCP1-R	GTGGGTTGCCCAATGAATAC	(3)
6	PRDM16-F	AGGACATTGAGCCAGGTGAG	
	PRDM16-R	GCTTGGACTGGAAGAGTTCG	

Table S4. Interaction of compounds with amino acid residues of PPARγ.

Protein	Compound	Binding Energy (kcal/mol)	H-Bond Interactions	Other Interactions	No. of H-Bond
**PPAR gamma**	Rutin	-8.2	GLU259, CYS285, SER342, ILE262	ILE281, GLY284, ARG288, ILE341	4
	CGA	-7.7	TYR327, SER289, CYS285, SER342	ARG288	4
	NCGA	-7.3	TYR327, SER289, GLY284	CYS285, MET364, SER342, LEU330	3
	CCGA	-7.2	SER342, SER289, CYS285	ARG288, MET364, LEU330	3
	Resveratrol	-6.9	CYS285, SER289	LEU330, ARG288, MET329, ALA292	2
	Rosiglitazone	-7.5	GLU343, ARG288, LYS265	ILE281, ARG280, LEU255, HIS266	3

Table S5. Active site prediction for PPARγ and UCP1 using DoGSiteScorer.

Protein	Site	Volume Å^3	Surface Å^2	Drug Score	Simple Score
**PPARγ**	S1	2214.45	2608.88	0.81	0.66
	S2	2095.07	2287.47	0.82	0.67
	S3	186.76	275.29	0.45	0.04
**UCP1**	S1	603.52	1043.49	0.81	0.45
	S2	504.96	794.53	0.64	0.42
	S3	194.18	352.06	0.27	0.09

Table S6. Interaction of compounds with amino acid residues of UCP1.

Protein	Compound	Binding Energy (kcal/mol)	H-Bond Interactions	Other Interactions	No. of H-Bond
**UCP1**	Rutin	-8.4	LYS73, ARG84, THR36, GLN144, HIS146, GLN142, LEU141, LEU147	HIS148, GLY77, PHE32, VAL65	8
	CGA	-6.8	GLN85, ASP35	PHE32, ARG84	2
	NCGA	-7.2	ARG84, GLN85, LYS73	SER76, PHE32	3
	CCGA	-7.1	ARG84, GLN142	PHE32	2
	Resveratrol	-7.4	LYS73, SER76, GLN85, GLN142	GLY77, PHE32	4
	Rosiglitazone	-7.2	ARG84	PHE32, VAL65	1

Table S7. Parameters evaluated for drug-likeness.

Compound	MCE-18	SAscore	Fsp^3	PAINS	NPScore	Pfizer
**Rutin**	122.949	4.783	0.444	1	2.015	Accepted
**CGA**	60.273	3.871	0.375	1	2.246	Accepted
**NCGA**	60.273	3.871	0.375	1	2.246	Accepted
**CCGA**	60.273	3.585	0.375	1	1.98	Accepted
**Rosiglitazone**	16.0	2.501	0.222	0	-1.175	Accepted
**Resveratrol**	11.0	2.112	0.0	0	0.754	Accepted

Table S8. Parameters evaluated for absorption.

Compound	Caco-2 permeability	MDCK permeability	Pgp-inhibitor	Pgp-substrate	HIA	F (20%)	F (30%)
**Rutin**	-6.336	3e-05	0.002	0.978	0.925	0.234	0.999
**CGA**	-6.127	9.9e-05	0.0	0.558	0.873	0.822	0.996
**NCGA**	-6.127	9.9e-05	0.0	0.558	0.873	0.822	0.996
**CCGA**	-6.257	0.000124	0.0	0.628	0.939	0.985	0.999
**Rosiglitazone**	-4.878	2.8e-05	0.001	0.001	0.013	0.142	0.073
**Resveratrol**	-4.916	1.4e-05	0.455	0.102	0.012	0.264	0.055

Table S9. Parameters evaluated for distribution.

Compound	PPB (%)	VD (L/kg)	BBB (log BB)	Fu (%)
**Rutin**	83.81%	0.754	0.111	20.86%
**CGA**	67.18%	0.351	0.59	34.06%
**NCGA**	67.18%	0.351	0.59	34.06%
**CCGA**	42.26%	0.457	0.791	47.09%
**Rosiglitazone**	92.04%	0.475	0.137	3.157%
**Resveratrol**	97.26%	0.822	0.032	2.620%

Table S10. Parameters evaluated for metabolism.

Compound	**CYP1A2 inhibitor**	**CYP1A2 substrate**	**CYP2C19 inhibitor**	**CYP2C19 substrate**	**CYP2C9 inhibitor**	**CYP2C9 substrate**	**CYP2D6 inhibitor**	**CYP2D6 substrate**	**CYP3A4 inhibitor**	**CYP3A4 substrate**
**Rutin**	0.013	0.026	0.011	0.05	0.002	0.246	0.007	0.155	0.013	0.003
**CGA**	0.036	0.042	0.023	0.053	0.016	0.511	0.003	0.156	0.03	0.022
**NCGA**	0.036	0.042	0.023	0.053	0.016	0.511	0.003	0.156	0.03	0.022
**CCGA**	0.168	0.039	0.012	0.047	0.006	0.078	0.014	0.122	0.107	0.036
**Rosiglitazone**	0.789	0.503	0.964	0.065	0.942	0.582	0.743	0.524	0.814	0.798
**Resveratrol**	0.976	0.11	0.229	0.056	0.356	0.957	0.629	0.915	0.943	0.163

Table S11. Parameters evaluated for excretion.

Compound	CL	$T_{1/2}$
**Rutin**	1.349	0.524
**CGA**	3.251	0.928
**NCGA**	3.251	0.928
**CCGA**	2.49	0.922
**Rosiglitazone**	8.793	0.475
**Resveratrol**	15.661	0.924

Table S12. Parameters evaluated for toxicity.

Compound	hERG Blockers	H-HT	DILI	AMES Toxicity	ROA	Skin Sensitization	Carcinogenicity	Eye Corrosion	Respiratory Toxicity
**Rutin**	0.017	0.092	0.982	0.805	0.05	0.036	0.064	0.003	0.015
**CGA**	0.014	0.122	0.037	0.026	0.028	0.156	0.059	0.003	0.031
**NCGA**	0.014	0.122	0.037	0.026	0.028	0.156	0.059	0.003	0.031
**CCGA**	0.02	0.122	0.032	0.029	0.047	0.519	0.061	0.003	0.05
**Rosiglitazone**	0.04	0.642	0.986	0.316	0.048	0.058	0.673	0.003	0.021
**Resveratrol**	0.109	0.374	0.032	0.076	0.451	0.959	0.287	0.047	0.405

References

1. Simu, S.Y., Siddiqi, M.H., Ahn, S., Castro-Aceituno, V., Kumar, N.S., Perez, Z.E.J. and Yang, D.C., 2017. Ginsenoside F1 attenuates lipid accumulation and triglycerides content in 3T3-L1 adipocytes with the modulation of reactive oxygen species (ROS) production through PPAR-γ/JAK2 signaling responses. *Medicinal Chemistry Research*, *26*(5), pp.1042-1051.
2. Siraj, F.M., SathishKumar, N., Kim, Y.J., Kim, S.Y. and Yang, D.C., 2015. Ginsenoside F2 possesses anti-obesity activity via binding with PPARγ and inhibiting adipocyte differentiation in the 3T3-L1 cell line. *Journal of enzyme inhibition and medicinal chemistry*, *30*(1), pp.9-14.
3. Li, H., Zhang, Y., Wang, F., Donelan, W., Zona, M.C., Li, S., Reeves, W., Ding, Y., Tang, D. and Yang, L., 2019. Effects of irisin on the differentiation and browning of human visceral white adipocytes. *American journal of translational research*, *11*(12), p.7410.
